# Doubly resonant enhancement of second-harmonic generation with in-plane phase matching in plasmonic metasurfaces on an AlInP slab waveguide

**DOI:** 10.1039/d6na00596a

**Published:** 2026-10-21

**Authors:** Timo Stolt, Huayu Bai, Seyed Ahmad Shahahmadi, Jani Oksanen, Andriy Shevchenko, Radoslaw Kolkowski

**Affiliations:** a Department of Applied Physics, Aalto University P.O. Box 13500 FI-00076 Aalto Finland andriy.shevchenko@aalto.fi radoslaw.kolkowski@aalto.fi; b Engineered Nanosystems Group, Aalto University P.O. Box 13500 FI-00076 Aalto Finland

## Abstract

Nonlinear metasurfaces have attracted significant interest by offering the possibility to circumvent conventional phase-matching requirements of bulk nonlinear crystals, opening the way to efficient frequency conversion over ultrashort propagation distances. In this context, one of the most promising and well-established strategies is to use the so-called nonlocal metasurfaces supporting spatially extended resonances with high quality (*Q*) factors. Here, we experimentally demonstrate metasurfaces in which second-harmonic generation is strongly enhanced by in-plane phase matching of high-*Q* guided-mode resonances. To achieve this enhancement, we use hybrid metasurfaces composed of periodic arrays of gold nanodiscs on an unpatterned slab waveguide made of aluminum indium phosphide (AlInP) – a low-loss epitaxial III–V semiconductor material. The metasurfaces are found to exhibit guided-mode resonances with *Q* factors exceeding 400 and 900 at visible and near-infrared wavelengths, respectively. Our frequency and momentum-resolved measurements demonstrate a two-orders-of-magnitude enhancement of second-harmonic generation by these high-*Q* modes under doubly resonant conditions. We reproduce the experimental results using numerical simulations, showing that the in-plane phase matching and spatial mode overlap are the main factors governing the enhancement. Our findings demonstrate a promising strategy to realize efficient metasurface-based frequency converters, enabling their potential applications in compact photonic systems.

## Abstract 2

This work demonstrates strong enhancement of nonlinear optical frequency conversion by guided-mode resonances in hybrid plasmonic-III–V-semiconductor metasurfaces.

## Graphical abstract

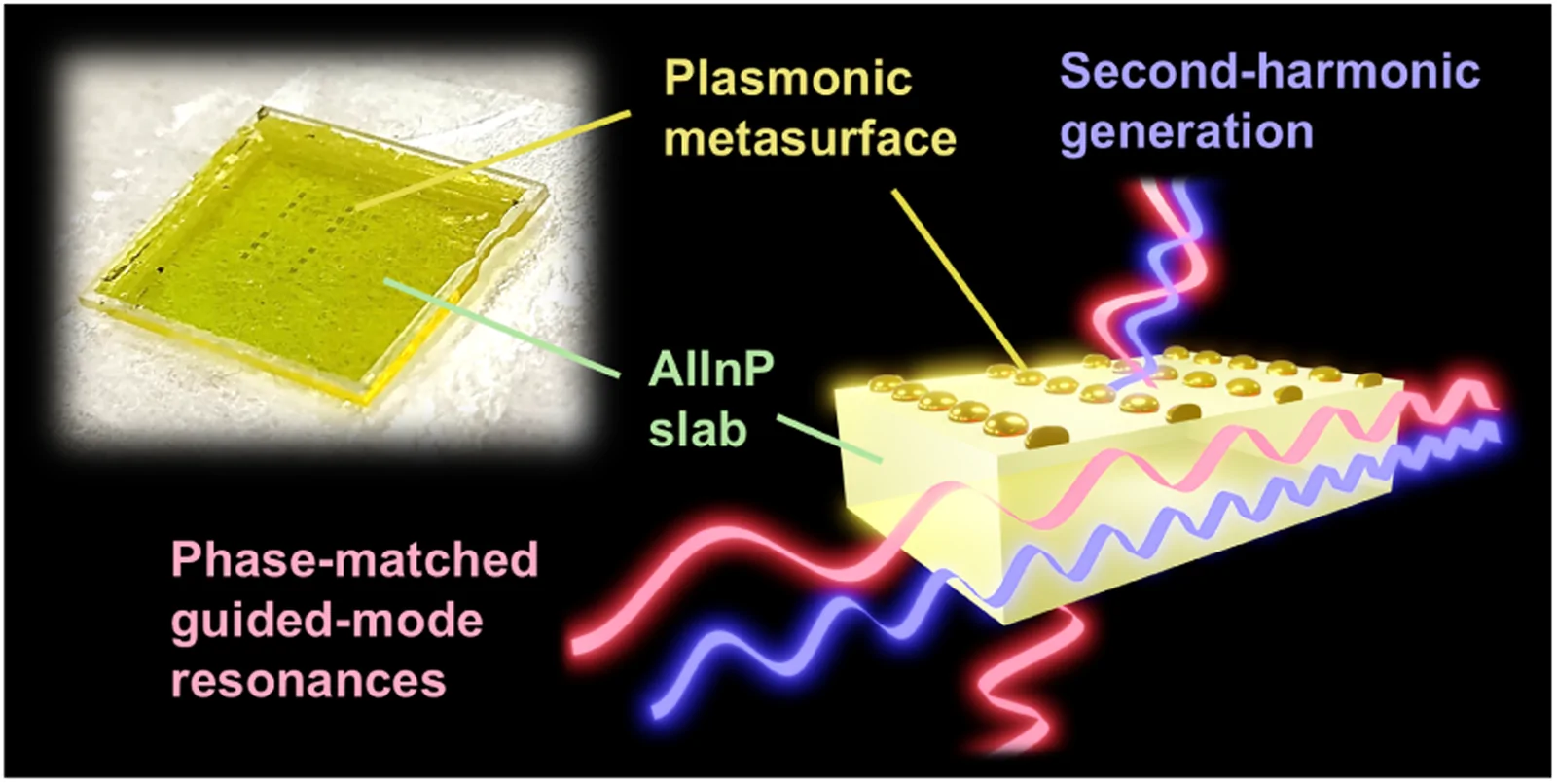

## 1 Introduction

Nonlinear optical processes are the cornerstone of many modern photonic applications, ranging from pulsed and wavelength-tunable lasers to photon pair generation for quantum technologies.^1,2^ A well-known example of such processes is second-harmonic generation (SHG), in which two photons with the same frequency are annihilated to generate a new photon with a doubled frequency. Consequently, SHG is often used to up-convert laser light from the near-infrared to the visible spectral range.

SHG arises from a material nonlinearity characterized by the second-order susceptibility *χ*^(2)^ inherent in non-centrosymmetric crystals, such as lithium niobate and beta-barium borate. However, in most of these crystals, the intrinsic nonlinear response is extremely weak. To enhance the response to a practical level, special phase-matching techniques are used. They allow minimization of the wavevector mismatch between the interacting light fields of different wavelengths. With phase matching achieved, the nonlinear frequency conversion shows a high energy conversion efficiency that can be further improved by placing the crystal in an optical resonator.^3^ Unfortunately, conventionally used phase matching can only be realized in birefringent media, excluding isotropic nonlinear materials, such as III–V semiconductors, which can possess high values of *χ*^(2)^.^4–7^ Therefore, in addition to phase matching using optical anisotropy, other methods have been used, such as quasi-phase matching in periodically poled crystals.^8–11^ However, to enable significant device miniaturization, one has to explore alternative approaches, aiming at efficient frequency conversion over much shorter propagation distances. One of such approaches is based on the use of optical metasurfaces.^12–15^

Metasurfaces are two-dimensional arrangements of nanoscale building blocks called meta-atoms, typically fabricated on a transparent substrate.^16,17^ Optically resonant meta-atoms, such as plasmonic nanoantennas and Mie-resonant dielectric particles, allow for resonant enhancement of light–matter interactions^18^ and efficient nanoscale phase control of optical fields.^19^ The local field enhancement provided by such resonant metasurfaces is especially useful for boosting the nonlinear optical response and frequency conversion, which have quadratic and higher-order dependence on the amplitudes of the interacting waves.^20,21^

Initially, research on nonlinear metasurfaces focused on metal-based plasmonic nanoantennas,^22–25^ but later it was extended to include all-dielectric Mie-type resonators.^26–28^ In dielectric meta-atoms, the ohmic losses are absent, but light trapping and enhancement remain limited. This limitation can be removed by the use of collective resonances in periodic structures, including guided-mode resonances (GMRs),^29,30^ surface lattice resonances (SLRs),^31,32^ and waveguide-plasmon polaritons (WPPs).^33–35^ Reducing the radiative losses of such resonances *via* destructive interference in the far-field can further improve their light-confinement capabilities, giving rise to the so-called dark modes^36,37^ or quasi-bound states in the continuum (qBIC).^38,39^ The dispersive nature of collective resonances (*i.e.*, dependence on both the wavelength and the incidence angle)^40^ makes them particularly suitable for simultaneous manipulation of the spectral and spatial-frequency components of the fields, governing the design and operation of the so-called nonlocal metasurfaces.^41–45^ Most importantly, collective resonances can possess high quality (*Q*) factors^46,47^ and provide large local field enhancement, which can be used to boost nonlinear optical effects.^48–77^ For example, high-*Q* nonlocal resonances have been used to significantly enhance third-harmonic generation^52^ and four-wave mixing^59^ in silicon metasurfaces, and to enable efficient SHG of a continuous-wave pump in metasurfaces made of second-order nonlinear materials, such as gallium phosphide^53^ and lithium niobate.^60^ Enhancement of SHG by two orders of magnitude using high-*Q* nonlocal resonances has been achieved in metasurfaces made of patterned 2D materials, such as hexagonal boron nitride (in the visible range)^56^ and 3R molybdenum disulfide (in the near-infrared range).^61^ Collective resonances (SLRs, WPPs, and GMRs) have enabled significant enhancement of SHG in plasmonic^62–64^ and waveguide-coupled metal–dielectric metasurfaces.^65–69^ One of the most promising strategies to enhance the nonlinear conversion efficiency is to use an unpatterned (etchless) layer of a nonlinear material acting as a waveguide coupled to a resonant periodic structure. This paradigm has been extensively explored in designs based on a lithium niobate layer coupled to a dielectric periodic structure, demonstrating strong enhancement of nonlinear optical effects by high-*Q* quasi-BICs.^70–77^

Most of the recently demonstrated high-*Q* nonlinear metasurfaces are made of virtually lossless dielectric materials that are only weakly nonlinear. On the other hand, highly nonlinear materials, such as III–V semiconductors, exhibit considerable absorption losses in the visible spectral range in which the second harmonic is typically generated, reducing the overall frequency conversion efficiency.^78^ In this work, we implement the paradigm of using an unpatterned nonlinear layer^70–77^ to realize a hybrid metasurface design^65–69^ that combines a metal and a nonlinear dielectric. Specifically, we use a wide-bandgap zinc blende-type III–V semiconductor, AlInP, as a nonlinear, optically transparent material having a significant second-order susceptibility^79^ and a wide transparency range from the near-infrared range down to 500 nm wavelength.^80–83^ A slab of this material, being covered with an array of resonant gold nanoantennas, provides guidance and confinement of light in the form of GMRs, with low intensity at the position of the meta-atoms reducing the losses *via* mode interference.^84,85^ Large on-resonance scattering cross sections of the plasmonic meta-atoms provide efficient coupling of the incident light to GMRs and local enhancement of the confined field. The transparency of AlInP covers both the fundamental and SHG wavelengths, enabling the realization of a condition for doubly resonant SHG enhancement. Being created by epitaxial growth and subsequent wafer bonding, AlInP forms a high-quality crystalline layer with a negligible surface roughness, which minimizes the scattering losses of the guided modes. Due to the above properties, our Au–AlInP metasurfaces support high-*Q* GMRs, which we use to enhance the intrinsic SHG response of the AlInP layer. Importantly, we achieve high *Q* factors not only in the near-infrared range (as frequently reported in the literature), but also in the visible spectral range (*Q* ≈ 430 at *λ* ≈ 555 nm), which was rarely obtained when employing metasurfaces based on plasmonic nanostructures. Finally, we also show that the in-plane phase matching and spatial overlap between the fields of GMRs at the fundamental and SHG wavelengths can give rise to a large SHG enhancement, significantly surpassing that obtained away from the doubly resonant condition. These properties are clearly revealed by frequency- and momentum-resolved nonlinear optical measurements and confirmed by rigorous numerical simulations. Our findings pave the way towards further advancements in the field of resonant nonlinear metasurfaces, bringing them closer to practical applications in compact nonlinear photonic devices.

## 2 Metasurface design and experimental results

To demonstrate the doubly resonant SHG enhancement by GMRs, we designed and fabricated metasurfaces with a structure shown in Fig. 1a. The metasurfaces are composed of gold nanodiscs arranged into rectangular arrays. Each nanodisc has a height of 40 nm and a diameter of 80 nm. We fabricated and studied several samples with different periods *P*_*y*_ along the *y*-axis. Here, we report the results obtained with two of these samples: sample A with *P*_*y*_ = 420 nm and sample B with *P*_*y*_ = 360 nm. The period along the *x*-axis is *P*_*x*_ = 150 nm in all the samples (see an example scanning electron microscopy image in Fig. 1b and additional images in the SI). The arrays were fabricated *via* electron-beam lithography (EBL) on the surface of a 400 nm thick AlInP slab waveguide, which was first grown epitaxially on a gallium arsenide (GaAs) wafer using metal–organic vapor phase epitaxy (MOVPE) and subsequently transferred onto a glass substrate through a wafer-bonding process. We measured the optical constants of AlInP ellipsometrically, confirming its high real part of the refractive index (*n* > 3 at *λ* < 1000 nm) and wide transparency range (the imaginary part of the refractive index *κ* is <0.02 for *λ* > 500 nm), making it suitable for efficient confinement of light through low-loss guided modes. Since the surface of AlInP was exposed by chemical wet etching, we also characterized its roughness using atomic force microscopy (AFM), confirming its excellent surface quality (see the SI for more details).

**Fig. 1:**
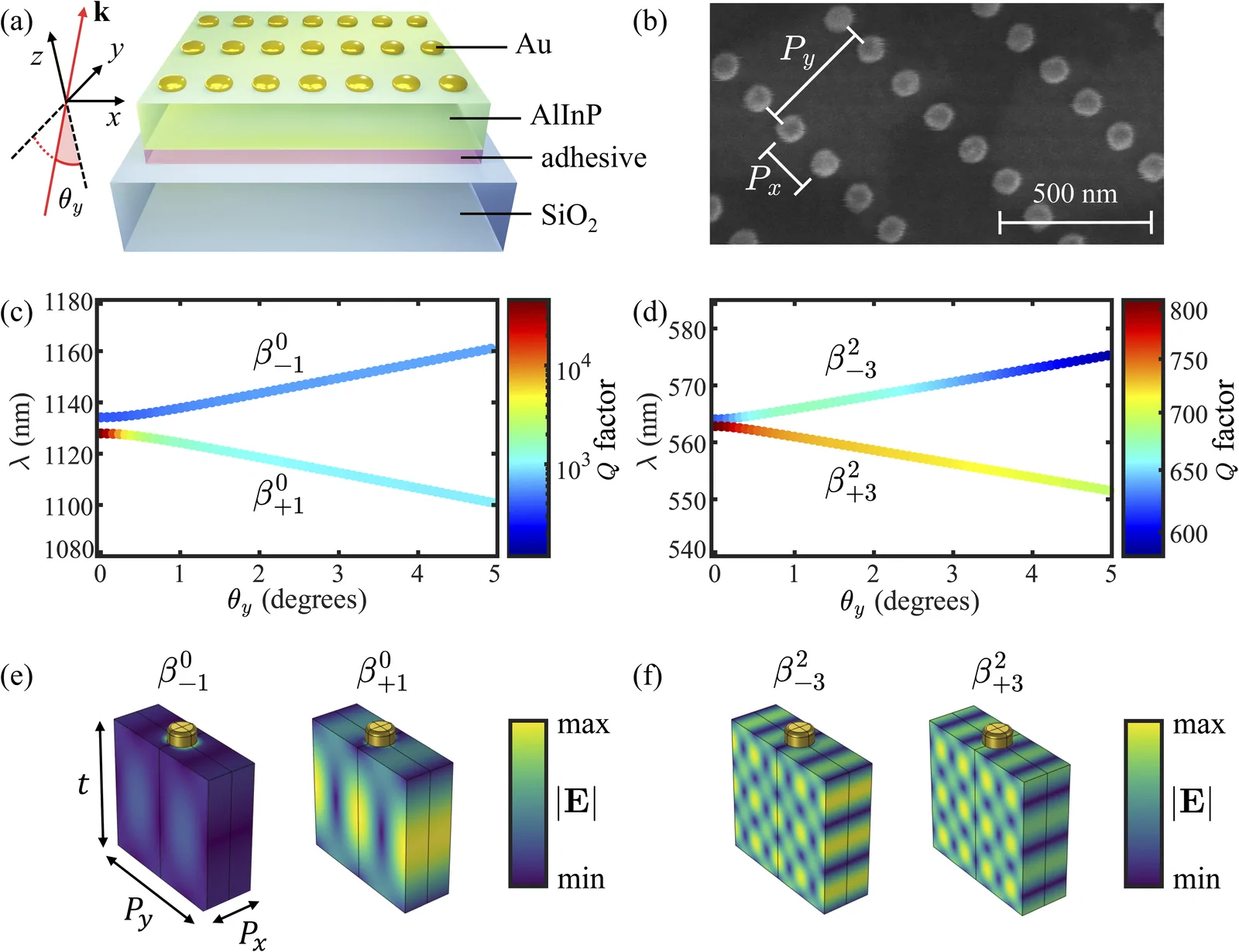
(a) Schematic illustration of a metal–dielectric metasurface consisting of a periodic array of gold nanodiscs deposited on top of an epitaxial AlInP waveguide, which is wafer-bonded to a glass substrate. The wavevector of incident/outgoing light (**k**) is illustrated with a red arrow, which shows its orientation in terms of the angle *θ*_*y*_ with respect to the Cartesian axes. (b) Example SEM image of the fabricated sample with periods *P*_*x*_ = 150 nm and *P*_*y*_ = 420 nm. Each nanodisc has a diameter of 80 nm and a thickness of 40 nm, while the thickness of the underlying AlInP layer is around 400 nm. (c–f) Results of the eigenfrequency analysis performed in COMSOL Multiphysics for different values of in-plane momentum (different points in *k*-space), expressed here in terms of the corresponding values of the incidence angle *θ*_*y*_ in the *yz*-plane. The analysis reveals high-*Q* transverse magnetic GMRs (with magnetic field **H** parallel to *x*) in the (c) infrared and (d) visible spectral ranges. The resonance *Q* factors are encoded in the colors of the eigenfrequency curves (see the color scales), reaching the highest values at the dark band-edge resonances (qBICs). (e and f) Distributions of the electric field norm |**E**| within one unit cell of the structure (with the glass substrate made invisible) at *θ*_*y*_ = 0 for the GMRs originating from the guided modes with propagation constants *β*_*m*_^*M*^ (*β*_*n*_^*N*^).

The period *P*_*x*_ of the metasurface is small, which makes the arrays dense, increasing the strength of their resonant response. For optical waves propagating along the *x*-axis, the arrays are sub-diffractive, which means that GMRs are primarily tailored by *P*_*y*_ through diffraction along the *y*-axis. In our samples, the (001) crystallographic plane of AlInP coincides with the *xy*-plane of the metasurface, while the [100] crystallographic direction is oriented at 45° with respect to the *x*-axis. In such a configuration, the GMRs originating from transverse-magnetic (TM) guided modes propagating along the *y*-axis can undergo nonlinear interactions through nonzero tensor elements *χ*_*yyz*_^(2)^, *χ*_*yzy*_^(2)^, and *χ*_*zyy*_^(2)^ (see the SI and the illustration in Fig. 3f). Due to the isotropic nature of the *χ*^(2)^ tensor in AlInP, the nonlinear interaction strength cannot be significantly improved by changing the crystal lattice orientation, as opposed to birefringent materials such as lithium niobate, in which the *χ*^(2)^ tensor typically has one dominant term which should be aligned with the nonlinearly interacting electric field vector components. The main purpose of this work is to demonstrate a specific enhancement mechanism rather than to reach the highest possible conversion efficiency. Hence, we choose a relatively simple configuration with GMRs having the same polarization (TM) and interacting only along one direction (*y*). The conversion efficiency could possibly be improved in more complex configurations, *e.g.*, using nonlinear interaction between the fundamental TE and second-harmonic TM modes.

Excitation of GMRs is governed by their phase matching to the incident wave as

1 *β*_*m*_^*M*^(*λ*_1_, *θ*_*y*1_) = *k*_*y*_(*λ*_1_, *θ*_*y*1_),

where integers *M* and *m* indicate the orders of the guided mode and diffraction, respectively, *λ*_1_ and *θ*_*y*1_ are the wavelength of the incident beam and its incidence angle in the *yz*-plane, respectively, *β*_*m*_^*M*^(*λ*_1_, *θ*_*y*1_) is the propagation constant of the guided Bloch mode, and *k*_*y*_(*λ*_1_, *θ*_*y*1_) is the *y*-component of the incident wavevector. The incident light is a collimated beam that initially propagates in air. Its in-plane wavevector component can be written as *k*_*y*_(*λ*_1_, *θ*_*y*1_) = sin(*θ*_*y*1_)2π/*λ*_1_.

Under the doubly resonant condition, the far-field radiation from a second-harmonic GMR is governed by

2 *β*_*n*_^*N*^(*λ*_2_, *θ*_*y*2_) = *k*_*y*_(*λ*_2_, *θ*_*y*2_),

where *β*_*n*_^*N*^(*λ*_2_, *θ*_*y*2_) is the propagation constant of the second-harmonic Bloch mode with orders *N* and *n*. Assuming that the SHG photons are emitted at the same angle at which the pump beam is incident on the other side of the metasurface (*θ*_*y*1_ = *θ*_*y*2_ = *θ*_*y*_) and using the relation *λ*_2_ = *λ*_1_/2, we obtain

3 *k*_*y*_(*λ*_2_, *θ*_*y*_) = sin(*θ*_*y*_)2π/*λ*_2_ = 2 sin(*θ*_*y*_)2π/*λ*_1_ = 2*k*_*y*_(*λ*_1_, *θ*_*y*_).

This means that, under the doubly resonant condition, the phase matching requirement

4 Δ*β* = *β*_*n*_^*N*^(*λ*_2_, *θ*_*y*_) − 2*β*_*m*_^*M*^(*λ*_1_, *θ*_*y*_) = *k*_*y*_(*λ*_2_, *θ*_*y*_) − 2*k*_*y*_(*λ*_1_, *θ*_*y*_) = 0

is automatically satisfied.

The metasurfaces are designed to enable doubly resonant SHG enhancement for fundamental wavelengths (*λ*_1_) around 1100 nm and SH wavelengths (*λ*_2_) around 550 nm. In sample A (*P*_*y*_ = 420 nm), the eigenfrequency analysis reveals high-*Q* GMRs at near normal incidence, associated with the Bloch modes marked by their propagation constants *β*^0^_±1_ and *β*_±3_^2^ in Fig. 1c and d (in the subsequent text, we will refer to GMRs associated with the Bloch modes with propagation constants *β*_*m*_^*M*^ briefly as “*β*_*m*_^*M*^ GMRs”). Their dispersion curves for the positive and negative diffraction orders anti-cross at *θ*_*y*_ = 0 in both wavelength ranges (near 1130 nm and 562 nm, respectively††Due to the dispersion of optical constants, the exact values of the resonant wavelengths obtained by the eigenfrequency analysis depend slightly on the simulation settings, *e.g.*, the frequency around which the eigenmodes are searched for.), giving rise to bright and dark band-edge resonances. In the near-infrared range, the bright resonance corresponds to a GMR standing wave with nodes located at the positions of the nanodiscs. This is characterized by a high magnitude of the *y*-component of the electric field at the nanodiscs (see the left plot in Fig. 1e). In the case of the dark resonance (qBIC), the evanescent field is primarily polarized in the *z*-direction, avoiding strong interaction with the nanodiscs and reducing the ohmic and radiation losses simultaneously.^85^ In the visible range, the situation is reversed, *i.e.*, the lower-*Q* bright mode corresponds to the nanodisc at the anti-node of the GMR. This difference can be attributed to the short period of the standing wave, which becomes comparable to the lateral size of the nanodisc, and the fact that the gold nanodisc is a less efficient scatterer at shorter wavelengths. The eigenfrequency analysis shows that the dark GMRs exhibit very high *Q* factors near *θ*_*y*_ = 0, on the order of 10^4^ and 10^3^ in the spectral ranges of the fundamental and SH waves, respectively.

Experimentally, GMRs in question are visible as narrow lines in the transmission of *y*-polarized light (see Fig. 2a and b), which we measured as a function of the wavelength and incidence angle *θ*_*y*_ (in Fig. 2a and b, the GMRs reduce/increase the transmittance contributing more to the reflected/transmitted light). The measurements were performed using a spectrally resolved back-focal-plane imaging setup, with a quartz tungsten-halogen lamp serving as the light source, a galvo mirror used for scanning the *k*-space (in combination with an aspheric lens and a high-NA objective), and an imaging spectrometer utilized for signal detection (with the transmitted light collected by another high-NA objective, having its back focal plane imaged on the spectrometer's entrance slit; see the SI). These transmission measurements yield GMR intersections at *θ*_*y*_ = 0 at the wavelengths of 1125 nm and 556 nm for the fundamental and SH waves, respectively, which are close to the simulated values. The anti-crossing is not clearly resolved in these measurements, but the spectral widths of the GMRs can be coarsely estimated at Δ*λ* ≈ 1 nm, corresponding to the *Q* factors of about 1000 and 500 in the infrared and visible spectral ranges, respectively. In addition to TM-polarized GMRs, our samples also exhibit transverse-electric (TE) GMRs, but we have found that they do not play a significant role in the SHG enhancement in the configuration used in our experiments, and therefore, we do not discuss them here. Their transmission spectra are shown in the SI.

**Fig. 2:**
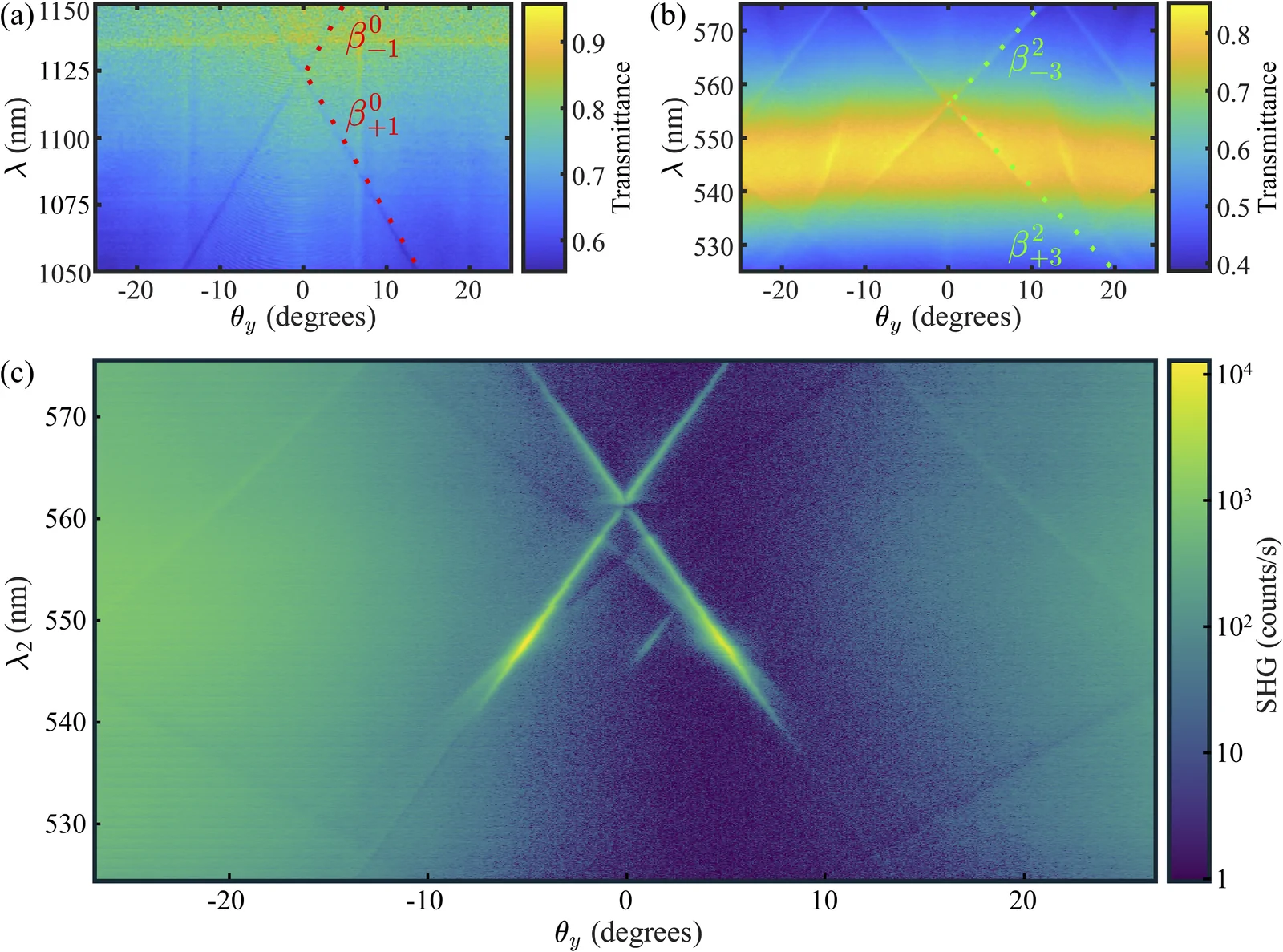
(a and b) Transmission of collimated TM-polarized light from a broadband incoherent source, measured as a function of the wavelength and incidence angle *θ*_*y*_ for sample A (*P*_*y*_ = 420 nm) in the (a) infrared and (b) visible spectral ranges. Red and green dotted lines highlight the GMRs that contribute to the measured SHG enhancement presented in (c).

Having established that sample A exhibits high-*Q* GMRs in the appropriate spectral regions, we subsequently measured its SHG emission as a function of the SH wavelength and emission angle (see Fig. 2c). We illuminated the sample from one side with a *y*-polarized beam of a tunable femtosecond laser (see the SI), scanning the incidence angle (as in the linear transmission measurements) and collecting the SHG signal with an objective located on the other side of the sample. The SHG signal was passed through a dichroic mirror and a set of short-pass filters blocking the fundamental beam. To probe the GMRs, we tuned the fundamental wavelength *λ*_1_ in the range of 1050–1150 nm, corresponding to the SH wavelength *λ*_2_ in the range of 525–575 nm. The obtained two-dimensional map of the measured SHG signal is presented in Fig. 2c, showing clear enhancement at the GMRs. The region of the doubly resonant condition is magnified in Fig. 3a.

**Fig. 3:**
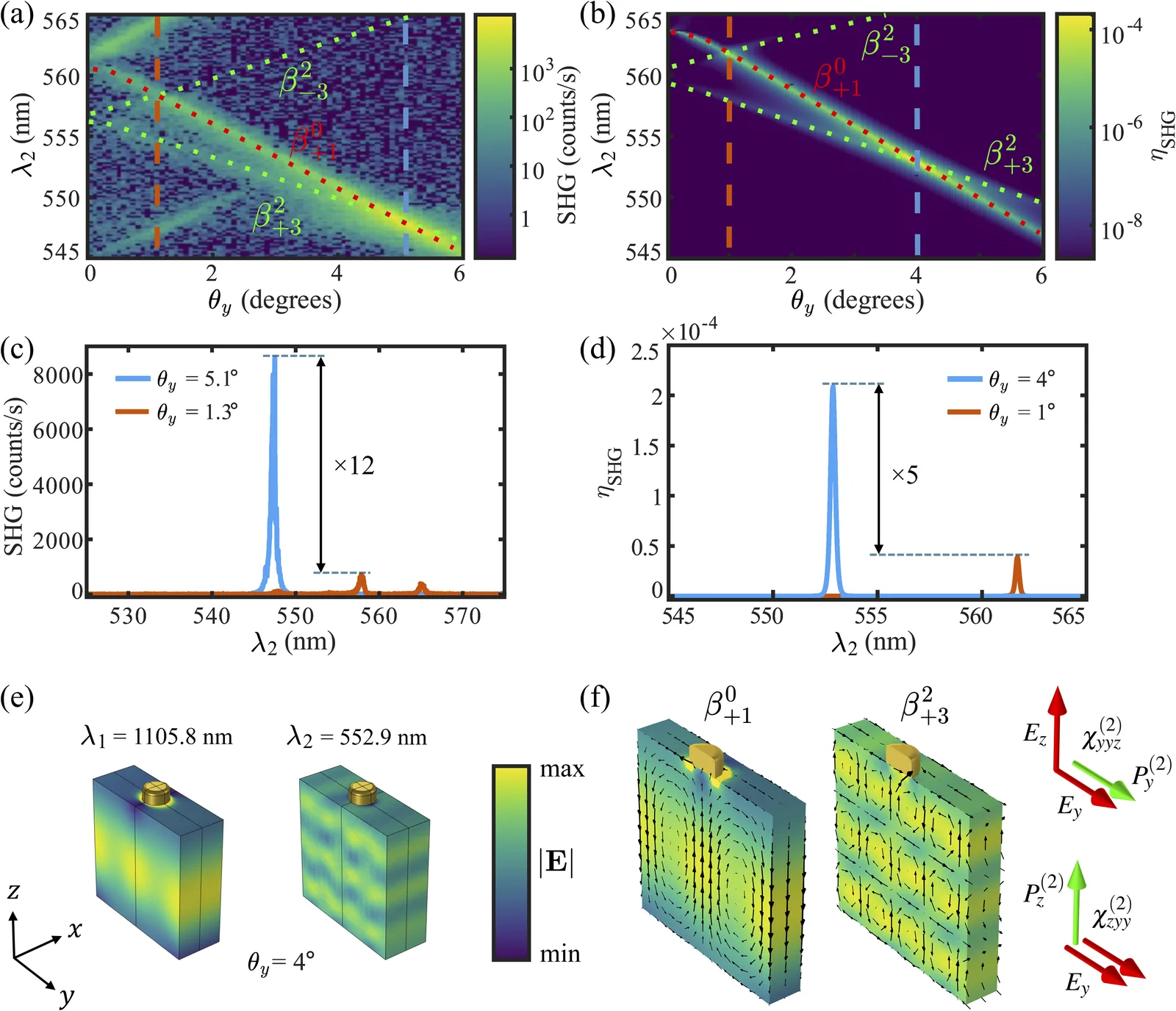
(a) Measured and (b) simulated dependence of the SHG on the wavelength (*λ*_2_) and incidence angle (*θ*_*y*_, equivalent to the emission angle) for sample A (*P*_*y*_ = 420 nm). The red dotted lines indicate the frequency-doubled dispersion of the *β*^0^_+1_ GMR, while the green dotted lines indicate the dispersion of the *β*_±3_^2^ GMRs. The orange and blue dashed lines highlight the doubly resonant conditions. Note that both SHG maps are plotted on a logarithmic color scale. The data and labels are presented separately in the SI to show some of the modes more clearly. (c) Measured and (d) simulated SHG spectra under the two doubly resonant conditions, indicated by the vertical dashed lines in (a) and (b). The spectra are plotted on a linear scale. (e) Simulated distributions of the electric-field norm |**E**| under the doubly resonant condition at *θ*_*y*_ = 4°, pump wavelength *λ*_1_ = 1105.8 nm, and SHG wavelength *λ*_2_ = 552.9 nm. (f) Simulated distributions of the electric-field norm |**E**| (color scale) and instantaneous electric-field vector **E** (black arrows) for the *β*^0^_+1_ and *β*_+3_^2^ modes at *θ*_*y*_ = 4°, obtained from the eigenfrequency analysis. Each of the plots shows only half of the unit cell, revealing the crosscut through its center. The red and green arrows on the right illustrate the vector components of the fundamental electric field and the nonlinear polarization density, respectively, coupled through the *χ*^(2)^-tensor elements specific for the crystallographic orientation of AlInP used in the experiments (see the SI).

To accurately identify the GMRs participating in the experimentally observed SHG enhancement, we modeled the SHG process *via* two-step full-wave frequency-domain scattering simulations in COMSOL Multiphysics (see the SI). The calculated far-field SHG power, expressed as the conversion efficiency *η*_SHG_, is presented in Fig. 3b. The simulated SHG map is in good agreement with the corresponding experimental results in Fig. 3a, except for a slight spectral shift of the GMR peaks. Moreover, the experimental data contain some additional features that are not reproduced by the simulations – these may result from the SHG processes that do not conserve the in-plane momentum (*e.g.*, due to scattering by non-periodic defects or reflection of the propagating Bloch modes from one of the edges of the array). Both experimental and theoretical maps show the highest SHG enhancement under the doubly resonant condition, where the frequency-doubled dispersion line of the *β*^0^_+1_ GMR at the pump wavelength (red dots) intersects with the dispersion line of the co-propagating *β*_+3_^2^ GMR at the SHG wavelength (green dots). This intersection occurs at *θ*_*y*_ = 5.1° in the experimental results and *θ*_*y*_ = 4° in the simulations (the angles are indicated by the vertical blue dashed lines in Fig. 3a and b). Another doubly resonant condition occurs at the intersection of the *β*^0^_+1_ GMR with the counter-propagating *β*_−3_^2^ GMR. This intersection is marked by the dashed orange lines at *θ*_*y*_ = 1.3°/1° in the experiment/simulation. The SHG enhancement by the *β*_−3_^2^ mode is relatively weak. To show it more clearly, we present another version of Fig. 3a and b in the SI, with the data and labels displayed separately. The measured and calculated SHG spectra in Fig. 3c and d show the vertical crosscuts through the two-dimensional maps along the vertical dashed lines in Fig. 3a and b. In Fig. 3e, we show the simulated electric field distributions at the pump and SHG wavelengths at *θ*_*y*_ = 4°. For comparison, Fig. 3f shows the corresponding results of the eigenfrequency analysis, revealing the distributions of the electric field for the *β*^0^_+1_ and *β*_+3_^2^ GMRs at *θ*_*y*_ = 4°. We also illustrate their coupling to the *χ*_*yyz*_^(2)^ (*χ*_*yzy*_^(2)^) and *χ*_*zyy*_^(2)^ tensor elements (see the red and green arrows in Fig. 3f on the right). The presented results confirm that the SHG enhancement indeed involves the *β*^0^_+1_ and *β*_+3_^2^ GMRs.

In doubly resonant structures, the total enhancement of the far-field SHG power 
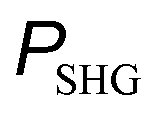
 depends on the local field enhancement factors *L*_1_ and *L*_2_ at the fundamental and SH wavelengths^86,87^ and the spatial overlap *Γ* of the fundamental and SH fields:^88–94^5
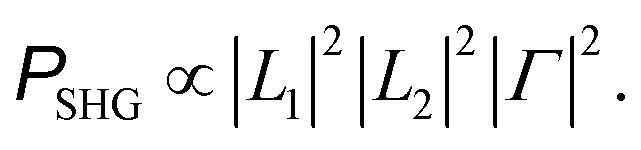
Note that *L*_1_ and *L*_2_ are defined for specific excitation conditions, which means that they take into account the efficiency of coupling between the GMRs and the far-field radiation (*i.e.*, excitation by the pump in *L*_1_ and SHG emission in *L*_2_). Eqn (5) shows that the SHG far-field power scales as the fourth power of the local field enhancement at the pump wavelength (*L*_1_), which explains the strong contribution of the *β*^0^_+1_ mode. In contrast, the local field enhancement at the SHG wavelength (*L*_2_) contributes only with its second power, which makes the contributions of the *β*_±3_^2^ modes significantly less pronounced in the SHG maps.

The most interesting observation is that the doubly resonant condition at the *β*^0^_+1_–*β*_+3_^2^ curve intersection gives rise to a much stronger SHG enhancement than at the *β*^0^_+1_–*β*_−3_^2^ intersection in both the experimental and theoretical results. In the experimental data (Fig. 3c), the maximum enhancement at *θ*_*y*_ = 5.1° (*β*^0^_+1_–*β*_+3_^2^ intersection) is about 12 times larger than the maximum enhancement at *θ*_*y*_ = 1.3° (*β*^0^_+1_–*β*_−3_^2^ intersection). The difference in the enhancement under the two doubly resonant conditions in the theoretical curves in Fig. 3d is slightly smaller (a factor of 5, compared to 12 in the experiment), but it is still remarkable. Note that these values are the ratios between the SHG enhancement factors, not their absolute magnitudes. Therefore, there is no reason to expect a higher value for the theoretical ratio. Despite the observed discrepancy between the exact values, the theory and experiments clearly show the same qualitative trend in the doubly resonant enhancement.

The above observations can be explained by investigating the mode overlap factor *Γ* (see eqn (5)), which in the general case can be expressed as^88–94^6
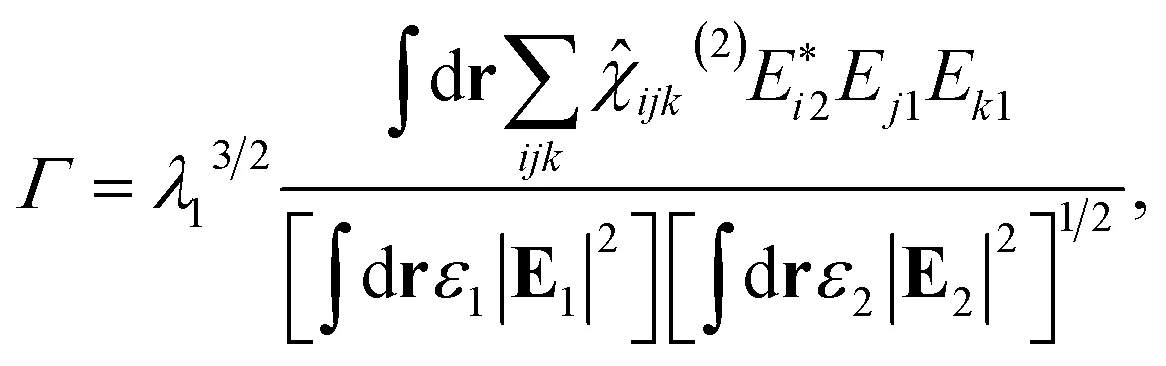
where 
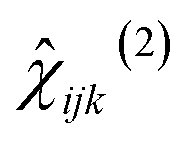
 are the normalized dimensionless elements of the second-order susceptibility tensor, *E*_*i*1_ and *E*_*i*2_ are the Cartesian electric field components of the fundamental and SH modes, respectively, *ε*_1_ and *ε*_2_ are the relative electric permittivities of the nonlinear material at the fundamental and SH wavelengths, respectively, and the integrals are calculated over the volume of the nonlinear material. In our case, we can safely assume that the measured SHG originates primarily from the AlInP layer, within which the pump and SHG fields propagate and interact. Plasmonic nanoparticles can also contribute to the SHG signal through the bulk and surface *χ*^(2)^ nonlinearity of gold^24,95^ which can be enhanced by localized plasmon resonances and surface defects that break the inversion symmetry of the nanoparticles. However, this SHG contribution would be observed at all incidence and emission angles, including the normal direction. Since we do not observe such an emission, the contribution to the SHG of the plasmonic nanoparticles can be neglected. Considering the *χ*^(2)^-tensor symmetry of AlInP and the illumination conditions used in our experiment, the integrand in the numerator of eqn (6) can be expressed as7
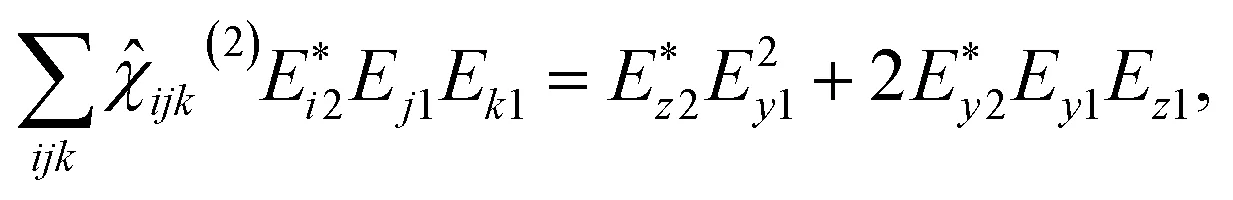
where 
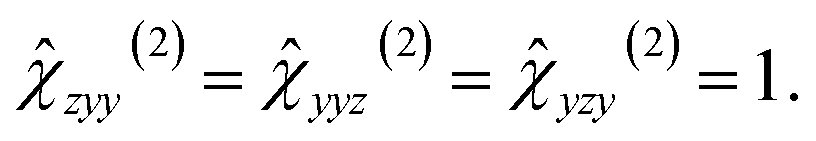
 To calculate the mode overlap factor *Γ* for the *β*^0^_+1_ and *β*_+3_^2^ modes under the doubly resonant condition, we calculated their electric field profiles using the eigenfrequency analysis at the *k*_*y*_ value that corresponds to *θ*_*y*_ = 4° (see Fig. 3f). Next, we calculated *Γ* for the *β*^0^_+1_ and *β*_−3_^2^ modes in the same way, setting *k*_*y*_ to that corresponding to *θ*_*y*_ = 1°. To make our comparison independent of the normalization method, we evaluated the ratio8
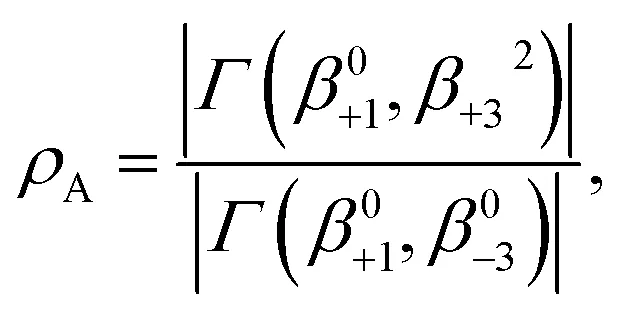
which is found to be equal to 5.1. This means that the overlap factor for the co-propagating *β*^0^_+1_ and *β*_+3_^2^ modes is five times larger than that for the counter-propagating *β*^0^_+1_ and *β*_−3_^2^ modes, which should lead to 25 times more efficient SHG. However, as can be seen in Fig. 1, the *β*_+3_^2^ mode has a higher *Q* factor (*i.e.*, smaller radiative loss) than the *β*_−3_^2^ mode. This means that the SHG radiation from the *β*_+3_^2^ mode is weaker than that from the *β*_−3_^2^ mode, which is why the SHG emission is enhanced by the mode overlap only 5 times in the simulations and 12 times in the experiment.

An additional factor that contributes to the experimentally observed SHG efficiency is the spatiotemporal walk-off of the fundamental and SH GMRs under femtosecond pulsed excitation. This walk-off depends on the relative group velocity, *i.e.*, the difference between the slopes of the dispersion curves of the frequency-doubled pump GMR and the SH GMR. Specifically, the walk-off may deteriorate the observed doubly resonant SHG enhancement for counter-propagating modes (such as *β*^0^_+1_ and *β*_−3_^2^) compared to the co-propagating modes (such as *β*^0^_+1_ and *β*_+3_^2^), which is consistent with our experimental observations. However, such spatiotemporal effects are not included in our simulations, which are performed in the frequency domain and take into account only one unit cell (*i.e.*, the system is assumed to be infinitely periodic in space and time). Furthermore, these effects are minimized in our experiments, as we illuminate the metasurfaces by plane waves that are uniform over a large area.

To estimate the *Q* factors of the GMRs, we analyzed the spectral widths of the peaks at *λ*_2_ as shown in Fig. 3a and used the relation *Q* ≈ *λ*/Δ*λ*, where Δ*λ* is the full width at half maximum (FWHM). The analysis is presented in the SI. For *θ*_*y*_ in the range of 1.5–2.5°, the frequency-doubled GMR dispersion of the *β*^0^_+1_ mode is well isolated from the neighboring SH GMRs. Fitting the averaged GMR peak with a Lorentzian curve gives Δ*λ*_1_ ≈ 1.23 nm at *λ*_2_ in the range of 554.5–557 nm. This gives *Q* = *λ*_1_/Δ*λ*_1_ ≈ 900 for the *β*^0^_+1_ mode at the pump wavelength *λ*_1_ ≈ 1112 nm. For *θ*_*y*_ in the range of 0.5–1.5°, the isolated SHG *via* the *β*_−3_^2^ mode has an average Δ*λ*_2_ equal to 1.29 nm at *λ*_2_ in the range of 553.5–555.5 nm. This yields *Q* = *λ*_2_/Δ*λ*_2_ ≈ 430 for the *β*_−3_^2^ mode at the SH wavelength.

Next, we turn to sample B with *P*_*y*_ = 360 nm, in which doubly resonant conditions occur at much larger incidence angles (*θ*_*y*_ of around 20°). At such angles, the off-resonant SHG signal from the AlInP layer becomes sufficiently bright to be used as a reference in evaluating the absolute magnitude of the resonant enhancement. The measured transmission and SHG spectra are presented in Fig. 4. The transmission measurements in the pump-wavelength range reveal only one GMR, namely *β*^0^_−1_ (red dotted line in Fig. 4a), while at the SHG wavelengths, we find two GMRs at similar incidence angles, *β*^0^_+4_ and *β*_−3_^2^ (yellow and green dotted lines in Fig. 4b, respectively). The signatures of these GMRs can be seen in the measured SHG enhancement map (see Fig. 4c). The doubly resonant conditions are found at *θ*_*y*_ = 18° for the *β*^0^_−1_–*β*^0^_+4_ mode intersection and *θ*_*y*_ = 21° for the intersection of the *β*^0^_−1_ and *β*_−3_^2^ modes, marked with the orange and blue vertical dashed lines, respectively. Fig. 4d shows the corresponding spectral crosscuts. The maximum SHG enhancement achieved under the two doubly resonant conditions differs by a factor of 35, which is much larger than that for sample A. Furthermore, under the phase-matched doubly resonant condition at *θ*_*y*_ = 21°, the overall SHG enhancement with respect to the off-resonant SHG response reaches a staggering factor of 390. The corresponding theoretical results for sample B are presented in the SI. Remarkably, the simulations predict that the SHG enhancement under the doubly resonant condition achieved with the *β*^0^_−1_ and *β*_−3_^2^ modes is 200 times larger compared to the case dealing with the *β*^0^_−1_ and *β*^0^_+4_ modes. We also calculated the ratio between the mode overlap factors9
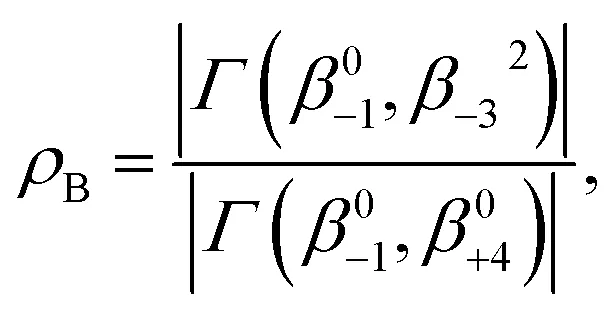
where *Γ*(*β*^0^_−1_, *β*_−3_^2^) and *Γ*(*β*^0^_−1_, *β*^0^_+4_) are calculated at *θ*_*y*_ = 21.2° and 17.2°, respectively. We find *ρ*_B_ to be equal to 33.2, which should give rise to a ∼1000 times greater SHG enhancement at the *β*^0^_−1_–*β*_−3_^2^ intersection compared to that at the *β*^0^_−1_–*β*^0^_+4_ intersection. The observed difference in the SHG enhancement is smaller (which, again, may be attributed to the different radiation efficiencies of the SH modes), but it agrees qualitatively with the corresponding ratio of the mode overlap factors. The results for both samples are summarized in Table 1, showing that the mode overlap correlates very strongly with the SHG enhancement in the studied systems.

**Fig. 4:**
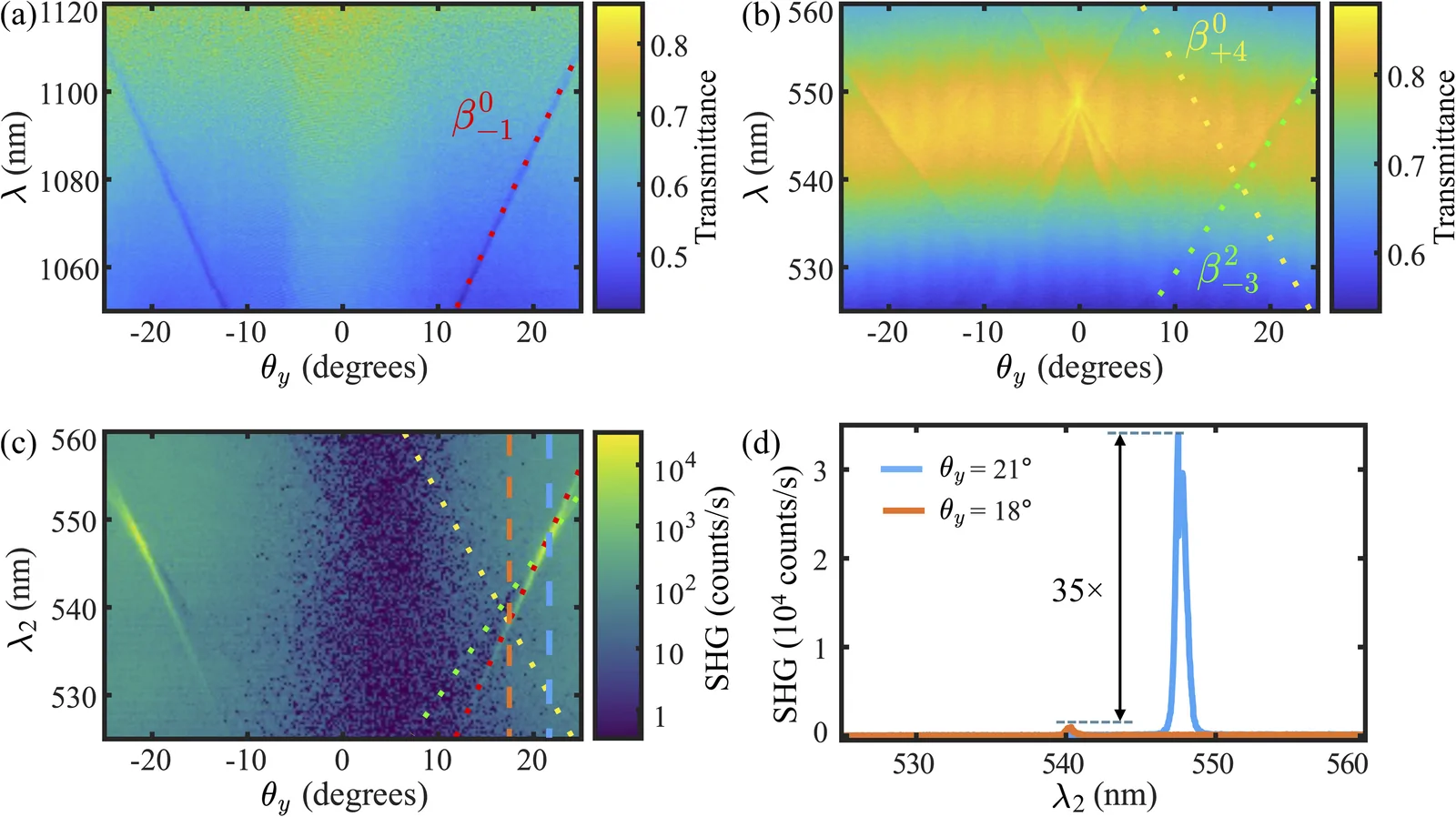
Experimental results for sample B (*P*_*y*_ = 360 nm). (a and b) Broadband transmittance measured as a function of incidence angle *θ*_*y*_ and wavelength in the (a) pump and (b) SHG spectral ranges. Red, green, and yellow dotted lines indicate the GMRs of interest. (c) SHG signal measured as a function of emission angle *θ*_*y*_ and SHG wavelength. Dotted lines follow the dispersion of the frequency-doubled GMR at the pump wavelengths (red) and higher-order GMRs at the SHG wavelengths (green and yellow). The angles at which doubly resonant conditions occur (18° and 21°) are marked with the orange and blue vertical dashed lines. (d) SHG spectra corresponding to the vertical crosscuts along the dashed lines in (c). The doubly resonant case at *θ*_*y*_ = 21° gives rise to 390-fold SHG enhancement with respect to the off-resonant SHG, which is 35 times larger than for the doubly resonant case at *θ*_*y*_ = 18°.

**Table 1:** Comparison of the SHG enhancement ratios and the mode overlap ratios under the doubly resonant conditions

Sample	*P*_*y*_ (nm)	GMRs	*λ*_2_ range (nm)	*θ*_*y*_ range (°)	SHG enhancement ratio		Ratio between the mode overlap factors
					Measured	Simulated	
A	420	*β* ^0^_+1_–*β*_+3_^2^*vs. β*^0^_+1_–*β*_−3_^2^	545–565	1–6	12	5	*ρ*_A_ = 5.1
B	360	*β* ^0^_−1_–*β*_−3_^2^*vs. β*^0^_−1_–*β*^0^_+4_	540–555	17–22	35	200	*ρ*_B_ = 33.2

## 3 Discussion

The above results provide interesting insights into the widely researched topic of nonlinear nonlocal metasurfaces, demonstrating that the efficiency of nonlinear optical effects in nonlocal metasurfaces can be greatly improved by simultaneous optimization of the phase matching and spatial overlap between guided-mode resonances at the wavelengths of the interacting waves. In more conventional “local” metasurfaces, nonlinear effects occur locally within the individual unit cells, so that their efficiency is primarily governed by the local-field enhancement and mode overlap, whereas the in-plane phase matching of the waves is not relevant. In our case, the nonlinear response originates from the resonant modes propagating in the plane of the metasurface and interacting with many unit cells simultaneously. Therefore, phase matching of the fundamental and second-harmonic GMR fields is crucial, while their spatial overlap determines further improvement of the SHG efficiency.

In addition to the doubly resonant enhancement of SHG, our results demonstrate the capability of hybrid plasmonic–dielectric metasurfaces to exhibit high-*Q* GMRs over a wide spectral range. To make optimal use of the doubly resonant conditions, the modes must have high *Q* factors at both fundamental and SH wavelengths. Achieving *Q* > 100 in the visible spectral range is often challenging with metasurfaces relying on plasmonic nanostructures. However, in our design, the light is confined mainly in the lossless AlInP waveguide, reducing the ohmic losses in gold nanodiscs. At the same time, the periodically distributed nanodiscs enable coupling between GMRs that results in the formation of dark modes (qBICs) with suppressed radiation loss in the anti-crossing regions (see Fig. 1c and d). The electric field distributions of these modes have minima that coincide with the locations of the nanodiscs, leading to further reduction of the ohmic losses (see Fig. 1e and f). The above mechanisms allow us to achieve very high *Q* factors even in the visible spectral range (*Q* ≈ 430 at *λ* ≈ 555 nm). An important aspect of our design is that it utilizes an unpatterned epitaxial layer of the nonlinear material acting as a waveguide,^70–77^ avoiding the scattering losses at inevitable imperfections of a patterned layer. Finally, the dispersive nature of GMRs allows the metasurfaces to be designed towards doubly resonant conditions at various wavelengths and incidence angles, which can be used to realize spectrally and directionally tunable frequency converters.

Last but not least, our results showcase AlInP as a promising material for nonlinear photonic devices. As a wide-bandgap III–V semiconductor possessing a noncentrosymmetric zinc blende crystal structure, AlInP has a considerable intrinsic nonlinearity (*d*_36_ = 39 pm V^−1^), while it is transparent over a wide spectral range (practically no absorption down to *λ* = 500 nm) and has a relatively high refractive index (*n* > 3) across the visible and near-infrared regions (see the SI for more details). The magnitude of the second-order susceptibility of AlInP is comparable to that of popular nonlinear photonic materials, such as lithium niobate (LN, *d*_33_ ≈ 30 pm V^−1^)^69^ and gallium phosphide (GaP, *d*_36_ ≈ 70 pm V^−1^).^53^ However, large anisotropy of the *χ*^(2)^ tensor in LN requires a different design approach to achieve efficient frequency conversion – in this case, the electric field vector must be aligned with the direction corresponding to the dominant *χ*^(2)^ tensor element.^69^ In contrast, in cubic materials such as AlInP and GaP, the crystal lattice orientation does not play such a critical role. From the point of view of the refractive index and transparency range, AlInP and GaP are similar, whereas LN has a significantly lower refractive index (2.2–2.3), which makes it less efficient in terms of confining light in photonic micro- and nanostructures. At the same time, the transparency range of LN is much wider than those of GaP and AlInP, extending to wavelengths as short as 350 nm. In view of the above performance tradeoffs, perhaps the most remarkable advantage of AlInP compared to LN and GaP is its fabrication feasibility. Indeed, AlInP is lattice-matched to GaAs, which allows AlInP thin films to be epitaxially grown on commercial GaAs wafers and subsequently transferred to transparent substrates *via* wafer bonding. In contrast, GaP is typically grown on another GaP substrate, whereas growing high-quality GaP thin films on different substrates (like Si and sapphire) is extremely challenging.^96^ Similarly, growing high-quality crystalline layers of LN and their subsequent transfer to lower-index substrates (LN-on-insulator platform) is an advanced process that cannot easily be implemented without a substantial technological know-how, forcing many researchers to rely on commercially supplied LN thin films.^97^ In summary, AlInP offers a decent tradeoff in terms of the second-order nonlinearity, refractive index, and transparency range, while also being suitable for relatively uncomplicated in-house epitaxial growth and thin-film fabrication. The above advantages of AlInP have so far been largely overlooked by the photonics community, which we hope will change after showcasing its potential in this work.

The specific approaches that we used to realize high-*Q* resonances and enhanced nonlinear optical effects can be adapted to a variety of different systems and material platforms. For example, gold nanodiscs could be replaced by other types of scatterers, such as Mie-resonant nanostructures or even air holes. Instead of TM modes, one can utilize TE-polarized GMRs to boost nonlinear effects excited at normal incidence, while the waveguide could be made of another low-loss nonlinear material. We also foresee that diverse multi-resonant and phase-matching scenarios could be realized to enhance other nonlinear frequency conversion processes, including sum-frequency generation and spontaneous parametric down-conversion. Such demonstrations could pave the way towards miniaturized counterparts of traditional nonlinear crystals used in wavelength-tunable lasers and photon-pair sources. Finally, we have demonstrated metasurfaces operating as free-space optical elements, but we believe that our design could also be adapted to photonic integrated circuits, opening new possibilities in nonlinear guided-wave manipulation.

## 4 Conclusions

In this work, we used hybrid plasmonic-dielectric metasurfaces to achieve doubly resonant enhancement of second-harmonic generation by high-*Q* guided-mode resonances, demonstrating that their phase matching and mode overlap play crucial roles in optimizing the nonlinear frequency conversion efficiency. In particular, we have demonstrated that optimizing the mode overlap between the phase-matched GMRs allows the SHG enhancement to be improved by a large factor (up to 35 in the experiments and up to 200 in the simulations), which allowed us to reach a total enhancement of 390 compared to the off-resonant SHG reference. These remarkable observations were enabled by the dispersive nature and high *Q* factors of the GMRs, which we achieved at both visible and near-infrared wavelengths (*Q* ≈ 430 at *λ* ≈ 555 nm and *Q* ≈ 900 at *λ* ≈ 1112 nm) despite the presence of lossy plasmonic nanoparticles, owing to the unique capabilities of hybrid systems to redistribute optical fields in a way avoiding losses. In addition, our results demonstrated AlInP as a promising III–V material for low-loss nonlinear photonics. Our approaches can be generalized to other material platforms, different nonlinear optical processes, and different systems including integrated photonic devices. Overall, our findings could pave the way towards highly compact, efficient, and tunable nonlinear optical components for both free-space and on-chip photonics.

## Conflicts of interest

There are no conflicts to declare.

## Supplementary Material

NA-OLF-D6NA00596A-s001

## Data Availability

All necessary data supporting the findings of this study are included in the article and its supplementary information (SI). Supplementary information: the description of methods (fabrication, experiments, and numerical simulations), additional results (SEM, AFM, ellipsometry, SHG power dependence, simulated results for sample B, and TE transmission spectra), and analysis (determination of the *χ*^(2)^ tensor elements and *Q* factors). Additional data and computational files are available on the Fairdata IDA platform at https://doi.org/10.23729/fd-c80a3f6e-dafb-3d98-9872-7000092dcc6f, ref. 98. See DOI: https://doi.org/10.1039/d6na00596a.

## References

[cit1] Boyd R. W. , Nonlinear Optics, Academic Press, San Diego, 2020

[cit2] Gerry C. C. and Knight P., Introductory Quantum Optics, Cambridge University Press, Cambridge, UK, New York, 2005

[cit3] Giordmaine J. A., Miller R. C. (1966). Appl. Phys. Lett..

[cit4] Anderson D., Boyd J. (1971). Appl. Phys. Lett..

[cit5] Somekh S., Yariv A. (1972). Appl. Phys. Lett..

[cit6] Fiore A., Berger V., Rosencher E., Bravetti P., Nagle J. (1998). Nature.

[cit7] Rivoire K., Lin Z., Hatami F., Masselink W. T., Vučković J. (2009). Opt. Express.

[cit8] Somekh S., Yariv A. (1972). Opt. Commun..

[cit9] Fejer M. M., Magel G. A., Jundt D. H., Byer R. L. (1992). IEEE J. Quantum Electron..

[cit10] Gordon L., Woods G., Eckardt R., Route R., Feigelson R., Fejer M., Byer R. (1993). Electron. Lett..

[cit11] Pantzas K., Combrié S., Bailly M., Mandouze R., Talenti F. R., Harouri A., Gérard B., Beaudoin G., Le Gratiet L., Patriarche G., De Rossi A., Léger Y., Sagnes I., Grisard A. (2022). ACS Photonics.

[cit12] Lee J., Tymchenko M., Argyropoulos C., Chen P.-Y., Lu F., Demmerle F., Boehm G., Amann M.-C., Alù A., Belkin M. A. (2014). Nature.

[cit13] Li G., Zhang S., Zentgraf T. (2017). Nat. Rev. Mater..

[cit14] Krasnok A., Tymchenko M., Alù A. (2018). Mater. Today.

[cit15] Kolkowski R., Hakala T. K., Shevchenko A., Huttunen M. J. (2023). Appl. Phys. Lett..

[cit16] Meinzer N., Barnes W. L., Hooper I. R. (2014). Nat. Photonics.

[cit17] Jahani S., Jacob Z. (2016). Nat. Nanotechnol..

[cit18] Koenderink A. F., Alù A., Polman A. (2015). Science.

[cit19] Yu N., Genevet P., Kats M. A., Aieta F., Tetienne J.-P., Capasso F., Gaburro Z. (2011). Science.

[cit20] Celebrano M., Wu X., Baselli M., Großmann S., Biagioni P., Locatelli A., Angelis C. D., Cerullo G., Osellame R., Hecht B., Duò L., Ciccacci F., Finazzi M. (2015). Nat. Nanotechnol..

[cit21] Minkov M., Gerace D., Fan S. (2019). Optica.

[cit22] Kauranen M., Zayats A. V. (2012). Nat. Photonics.

[cit23] Valev V. K., Baumberg J., De Clercq B., Braz N., Zheng X., Osley E., Vandendriessche S., Hojeij M., Blejean C., Mertens J., Biris C. G., Volskiy V., Ameloot M., Ekinci Y., Vandenbosch G. A. E., Warburton P. A., Moshchalkov V. V., Panoiu N. C., Verbiest T. (2014). Adv. Mater..

[cit24] Kolkowski R., Petti L., Rippa M., Lafargue C., Zyss J. (2015). ACS Photonics.

[cit25] Keren-Zur S., Avayu O., Michaeli L., Ellenbogen T. (2016). ACS Photonics.

[cit26] Liu S., Sinclair M. B., Saravi S., Keeler G. A., Yang Y., Reno J., Peake G. M., Setzpfandt F., Staude I., Pertsch T., Brener I. (2016). Nano Lett..

[cit27] Kivshar Y. (2018). Natl. Sci. Rev..

[cit28] Gigli C., Leo G. (2022). Opto-Electron. Adv..

[cit29] Quaranta G., Basset G., Martin O. J. F., Gallinet B. (2018). Laser Photonics Rev..

[cit30] Bej S., Tkachenko N., Fickler R., Niemi T. (2025). Adv. Opt. Mater..

[cit31] Wang W., Ramezani M., Väkeväinen A. I., Törmä P., Gómez Rivas J., Odom T. W. (2018). Mater. Today.

[cit32] Kravets V. G., Kabashin A. V., Barnes W. L., Grigorenko A. N. (2018). Chem. Rev..

[cit33] Christ A., Tikhodeev S., Gippius N., Kuhl J., Giessen H. (2003). Phys. Rev. Lett..

[cit34] Rodriguez S. R. K., Murai S., Verschuuren M., Gómez Rivas J. (2012). Phys. Rev. Lett..

[cit35] Zang X., Shevchenko A. (2023). New J. Phys..

[cit36] Rodriguez S. R. K., Abass A., Maes B., Janssen O. T., Vecchi G., Gómez Rivas J. (2011). Phys. Rev. X.

[cit37] Hakala T. K., Rekola H. T., Väkeväinen A. I., Martikainen J.-P., Nečada M., Moilanen A. J., Törmä P. (2017). Nat. Commun..

[cit38] Koshelev K., Lepeshov S., Liu M., Bogdanov A., Kivshar Y. (2018). Phys. Rev. Lett..

[cit39] Overvig A. C., Malek S. C., Carter M. J., Shrestha S., Yu N. (2020). Phys. Rev. B.

[cit40] Zhou W., Hua Y., Huntington M. D., Odom T. W. (2012). J. Phys. Chem. Lett..

[cit41] Overvig A. C., Malek S. C., Yu N. (2020). Phys. Rev. Lett..

[cit42] Overvig A., Alù A. (2022). Laser Photonics Rev..

[cit43] Chen Y., Fleury R., Seppecher P., Hu G., Wegener M. (2025). Nat. Rev. Phys..

[cit44] Monticone F., Mortensen N. A., Fernández-Domínguez A. I., Luo Y., Zheng X., Tserkezis C., Khurgin J. B., Shahbazyan T. V., Chaves A. J., Peres N. M., Wegner G., Busch K., Hu H., Della Sala F., Zhang P., Ciracì C., Aizpurua J. (2025). et al.. Opt. Mater. Express.

[cit45] Chen B., Reichler M., Kolkowski R., Shevchenko A. (2025). Phys. Rev. Appl..

[cit46] Zou S., Janel N., Schatz G. C. (2004). J. Chem. Phys..

[cit47] Bin-Alam M. S., Reshef O., Mamchur Y., Alam M. Z., Carlow G., Upham J., Sullivan B. T., Ménard J.-M., Huttunen M. J., Boyd R. W., Dolgaleva K. (2021). Nat. Commun..

[cit48] Kolkowski R., Szeszko J., Dwir B., Kapon E., Zyss J. (2016). Laser Photonics Rev..

[cit49] Huttunen M. J., Rasekh P., Boyd R. W., Dolgaleva K. (2018). Phys. Rev. A.

[cit50] Koshelev K., Tang Y., Li K., Choi D.-Y., Li G., Kivshar Y. (2019). ACS Photonics.

[cit51] Hooper D. C., Kuppe C., Wang D., Wang W., Guan J., Odom T. W., Valev V. K. (2019). Nano Lett..

[cit52] Liu Z., Xu Y., Lin Y., Xiang J., Feng T., Cao Q., Li J., Lan S., Liu J. (2019). Phys. Rev. Lett..

[cit53] Anthur A. P., Zhang H., Paniagua-Dominguez R., Kalashnikov D. A., Ha S. T., Maß T. W. W., Kuznetsov A. I., Krivitsky L. (2020). Nano Lett..

[cit54] Zhang X., He L., Gan X., Huang X., Du Y., Zhai Z., Li Z., Zheng Y., Chen X., Cai Y., Ao X. (2022). Laser Photonics Rev..

[cit55] Zograf G., Koshelev K., Zalogina A., Korolev V., Hollinger R., Choi D.-Y., Zuerch M., Spielmann C., Luther-Davies B., Kartashov D., Makarov S. V., Kruk S. S., Kivshar Y. (2022). ACS Photonics.

[cit56] Kühner L., Sortino L., Tilmann B., Weber T., Watanabe K., Taniguchi T., Maier S. A., Tittl A. (2023). Adv. Mater..

[cit57] Kolkowski R., Berkhout A., Roscam Abbing S. D. C., Pal D., Dieleman C. D., Geuchies J. J., Houtepen A. J., Ehrler B., Koenderink A. F. (2024). ACS Photonics.

[cit58] Weissflog M. A., Ma J., Zhang J., Fan T., Lung S., Pertsch T., Neshev D. N., Saravi S., Setzpfandt F., Sukhorukov A. A. (2024). Nanophotonics.

[cit59] Moretti G. Q., Weber T., Possmayer T., Cortés E., Menezes L. d. S., Bragas A. V., Maier S. A., Tittl A., Grinblat G. (2024). Nanophotonics.

[cit60] Di Francescantonio A., Sabatti A., Weigand H., Bailly-Rioufreyt E., Vincenti M. A., Carletti L., Kellner J., Zilli A., Finazzi M., Celebrano M., Grange R. (2025). Nat. Commun..

[cit61] Peng Z. H., Cotrufo M., Xu D., Mann S. A., Qiu S., Basov D., Delor M., Alú A., Schuck P. J., Trovatello C. (2025). Nat. Photonics.

[cit62] Michaeli L., Keren-Zur S., Avayu O., Suchowski H., Ellenbogen T. (2017). Phys. Rev. Lett..

[cit63] Stolt T., Vesala A., Rekola H., Karvinen P., Hakala T. K., Huttunen M. J. (2022). Opt. Express.

[cit64] Sharma M., Tal M., McDonnell C., Ellenbogen T. (2023). Sci. Adv..

[cit65] Abir T., Tal M., Ellenbogen T. (2022). Nano Lett..

[cit66] Ju Y., Zhang W., Zuo H. (2023). Photon. Nanostruct: Fundam. Appl..

[cit67] Li Z., Ye X., Hu Z., Li H., Liu S., Zheng Y., Chen X. (2024). Opt. Express.

[cit68] Abir T., Ellenbogen T. (2025). Nanophotonics.

[cit69] Ansimov D., Minerbi E., Tal M., Finazzi M., Celebrano M., Ellenbogen T. (2026). Opt. Mater. Express.

[cit70] Yang Q., Liu Y., Gan X., Fang C., Han G., Hao Y. (2020). IEEE Photonics J..

[cit71] Zhang J., Ma J., Parry M., Cai M., Camacho-Morales R., Xu L., Neshev D. N., Sukhorukov A. A. (2022). Sci. Adv..

[cit72] Cai S., Zong S., Liu X., Liu G., Chen J., Liu Z. (2023). Appl. Phys. Lett..

[cit73] Feng S., Liu T., Chen W., Wu F., Xiao S. (2023). Sci. China:Phys., Mech. Astron..

[cit74] Hu Y., Huang Z., Zeng X., Li Y., Luo K., Guan H., Cao Y., Lu H. (2023). Opt. Mater. Express.

[cit75] Valencia Molina L., Camacho Morales R., Zhang J., Schiek R., Staude I., Sukhorukov A. A., Neshev D. N. (2024). Adv. Mater..

[cit76] Qiu H., Tu X., Qin M., Wu F., Liu T., Xiao S. (2025). Phys. Rev. A.

[cit77] Hou Y., Luan Y., Fan Y., Nardi A., Zilli A., Du B., Shao J., Finazzi M., Wang C., Zhang L., Celebrano M. (2026). Laser Photonics Rev..

[cit78] Liu S., Vabishchevich P. P., Vaskin A., Reno J. L., Keeler G. A., Sinclair M. B., Staude I., Brener I. (2018). Nat. Commun..

[cit79] Shoji I., Kondo T., Ito R. (2002). Opt. Quant. Electron..

[cit80] Ochoa-Martínez E., Barrutia L., Ochoa M., Barrigón E., García I., Rey-Stolle I., Algora C., Basa P., Kronome G., Gabás M. (2018). Sol. Energy Mater. Sol. Cells.

[cit81] Kolkowski R., Shahahmadi S. A., Novikov S., Oksanen J., Liapis A. C., Bai H., Stolt T., Kaivola M., Shevchenko A. (2025). International Conference on Metamaterials, Photonic Crystals and Plasmonics (META).

[cit82] Kolkowski R. , Shahahmadi S. A., Novikov S., Oksanen J., Liapis A. C., Bai H., Stolt T., Kaivola M. and Shevchenko A., 2025 Nineteenth International Congress on Artificial Materials for Novel Wave Phenomena (Metamaterials), 2025, p. 176

[cit83] Kolkowski R. , Shahahmadi S. A., Novikov S., Oksanen J., Liapis A. C., Bai H., Stolt T., Kaivola M. and Shevchenko A., EPJ Web of Conferences, 2025, p. 06010

[cit84] Azzam S. I., Shalaev V. M., Boltasseva A., Kildishev A. V. (2018). Phys. Rev. Lett..

[cit85] Kolkowski R., Shevchenko A. (2023). Nanophotonics.

[cit86] Chen C. K., Heinz T. F., Ricard D., Shen Y. R. (1983). Phys. Rev. B:Condens. Matter.

[cit87] Maier S. A. , Plasmonics: Fundamentals and Applications, Springer US, New York, NY, 1st edn, 2007

[cit88] Rodriguez A., Soljačić M., Joannopoulos J. D., Johnson S. G. (2007). Opt. Express.

[cit89] O'Brien K., Suchowski H., Rho J., Salandrino A., Kante B., Yin X., Zhang X. (2015). Nat. Mater..

[cit90] Lin Z., Liang X., Lončar M., Johnson S. G., Rodriguez A. W. (2016). Optica.

[cit91] Minkov M., Gerace D., Fan S. (2019). Optica.

[cit92] Zanotti S., Minkov M., Fan S., Andreani L. C., Gerace D. (2021). Nanomaterials.

[cit93] Ge R., Liu X., Yan X., Chen X., Chen Y. (2023). Phys. Rev. B.

[cit94] Li L., Qu L., Wu W., Li C., Bai L., Wang C., Guo Z., Zhu P., Liu X., Cai W. (2026). et al.. Laser Photonics Rev..

[cit95] Bachelier G., Butet J., Russier-Antoine I., Jonin C., Benichou E., Brevet P.-F. (2010). Phys. Rev. B.

[cit96] Yang M., Weissflog M. A., Fedorova Z., Barreda A. I., Börner S., Eilenberger F., Pertsch T., Staude I. (2024). Nanophotonics.

[cit97] Saravi S., Pertsch T., Setzpfandt F. (2021). Adv. Opt. Mater..

[cit98] Kolkowski R. , Stolt T. and Bai H., Data for: Doubly resonant enhancement of second-harmonic generation with in-plane phase matching in plasmonic metasurfaces on an AlInP slab waveguide, Fairdata, 2026, 10.23729/fd-c80a3f6e-dafb-3d98-9872-7000092dcc6f

