# Supplementary material for: Doubly resonant enhancement of second-harmonic generation with in-plane phase matching in plasmonic metasurfaces on an AlInP slab waveguide
Source: Nanoscale Adv. 2026 Oct 21. Online ahead of print. doi: 10.1039/d6na00596a (PMC13753972; doi:10.1039/d6na00596a)
Supplement: Supplementary file 1 [file NA-OLF-D6NA00596A-s001.pdf]

Supplementary Information (SI) for Nanoscale Advances.
This journal is © The Royal Society of Chemistry

Supplementary Information for Doubly resonant enhancement of second-harmonic generation with in-plane phase matching in plasmonic metasurfaces on an AlInP slab waveguide

Timo Stolt,[a] Huayu Bai,[a] Seyed Ahmad Shahahmadi,[b] Jani Oksanen,[b]
Andriy Shevchenko,[a*] and Radoslaw Kolkowski[a*]

[a] *Department of Applied Physics, Aalto University, P.O.Box 13500, FI-00076 Aalto, Finland*
[b] *Engineered Nanosystems Group, Aalto University, P.O.Box 13500, FI-00076 Aalto, Finland*
* *E-mail: andriy.shevchenko@aalto.fi, radoslaw.kolkowski@aalto.fi*

Contents

I Sample fabrication 2

II Scanning electron microscopy images of the samples 2

III Atomic force microscopy of the AlInP surface 5

IV Ellipsometry results for the AlInP layer 6

V Experimental setup 8

VI Power dependence of the SHG signal 10

VII Data and labels from Fig. 3a-b shown separately 11

VIII Extracting the Q factor from the SHG map 11

IX Numerical simulations 13

X AlInP crystal orientation and the $\chi^{(2)}$ tensor 14

XI Simulated SHG response for sample B 15

XII TE-polarized guided-mode resonances 16

I Sample fabrication

A 400 nm thick AlInP layer was epitaxially grown on a GaAs wafer by metal–organic vapor phase epitaxy (MOVPE). The layer was then transferred onto a glass substrate via adhesive wafer bonding, followed by selective wet chemical etching[1] (with the help of etch-stop layers) to remove the GaAs substrate. The obtained AlInP layer bonded to glass was investigated by spectroscopic ellipsometry and atomic force microscopy (see Sections III and IV).

To fabricate the metasurfaces, a 200 nm thick PMMA resist was spin-coated on the AlInP surface and patterned by electron-beam lithography (EBL). The pattern was developed in a 1:3 MIBK:IPA solution. Next, a 40 nm thick Au film was deposited by electron-beam evaporation. Finally, a lift-off was performed in acetone for 4 hours without ultrasonic agitation or heating to avoid degradation of the used adhesive. The obtained samples were characterized using scanning electron microscopy (SEM; see Section II).

II Scanning electron microscopy images of the samples

Figures S1 and S2 show representative SEM images of samples A and B, respectively. The images were obtained at different magnifications, starting from the zoomed-out views of the whole metasurfaces (top left) and ending at the close-ups of the gold nanodiscs (bottom). At intermediate magnifications, the images reveal the high regularity of the lattice periods.

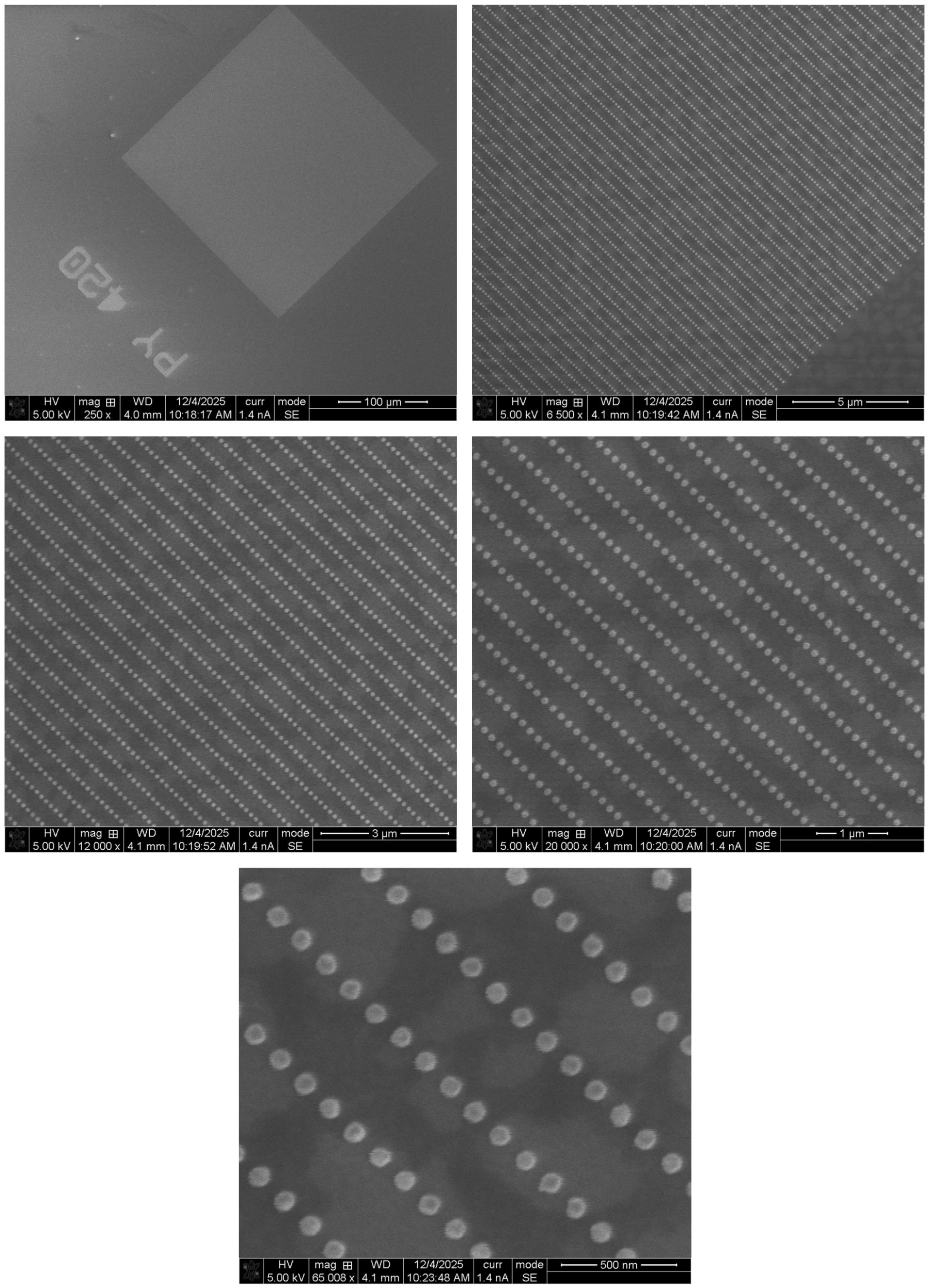

Figure S1 SEM images of sample A (P_y = 420 nm).

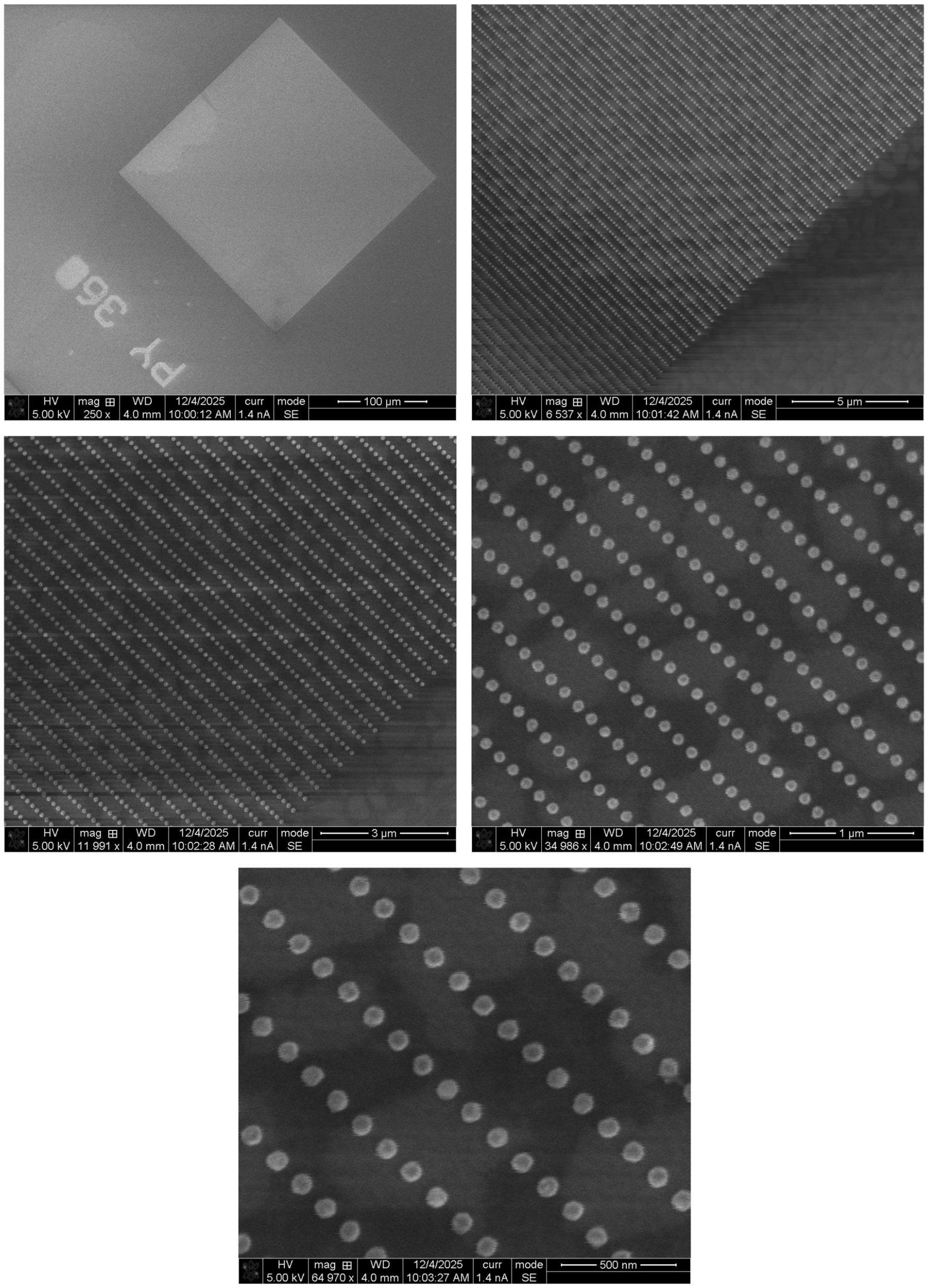

Figure S2 SEM images of sample B ($P_y = 360$ nm).

III Atomic force microscopy of the AlInP surface

We characterized the surface roughness of AlInP using atomic force microscopy (AFM). The results are shown in Fig. S3. The AlInP surface exposed by the chemical etching of GaAs appears to be relatively smooth. On the small scale (sub-100 nm in the lateral directions), the topography is dominated by shallow grains (height on the order of 1–2 nm), and on the larger scale ($\sim$1 µm), by branched ridges (height 5–10 nm).

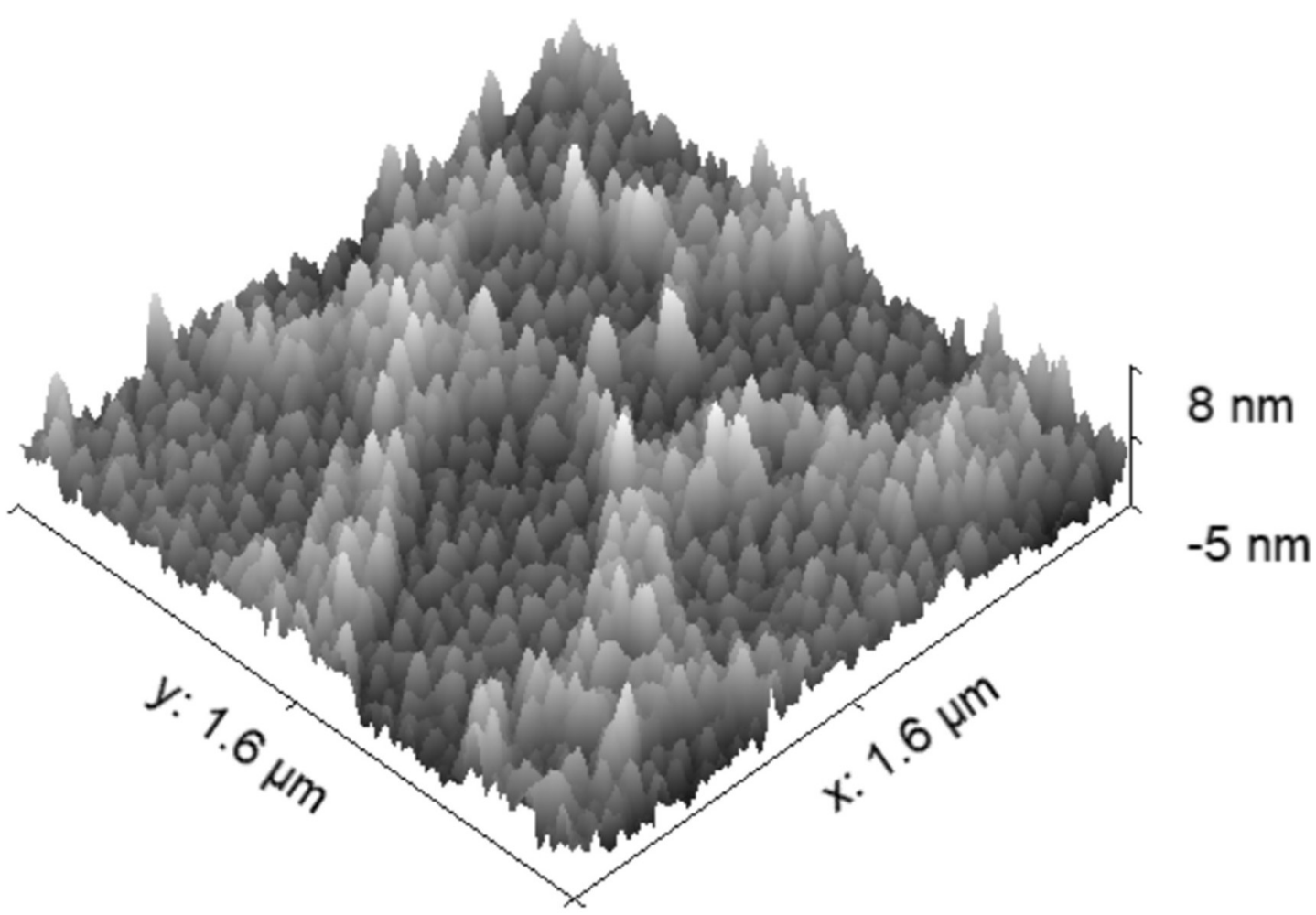

Figure S3 Surface roughness of AlInP measured by AFM. The mean roughness is 1.5 nm. The maximum peak height is 8.1 nm and the maximum pit depth is 5.2 nm.

IV Ellipsometry results for the AlInP layer

The optical properties of AlInP were studied using spectroscopic ellipsometry at variable incidence angles. The results of these measurements are presented in Fig. S4. The fitted optical constants (n, κ) are shown in Fig. S5 and their values are provided in Table S1. In addition, we studied the stability of the ellipsometric curves over six months. The results are presented in Fig. S6, showing that slow oxidation of AlInP has a very small effect on its optical properties.

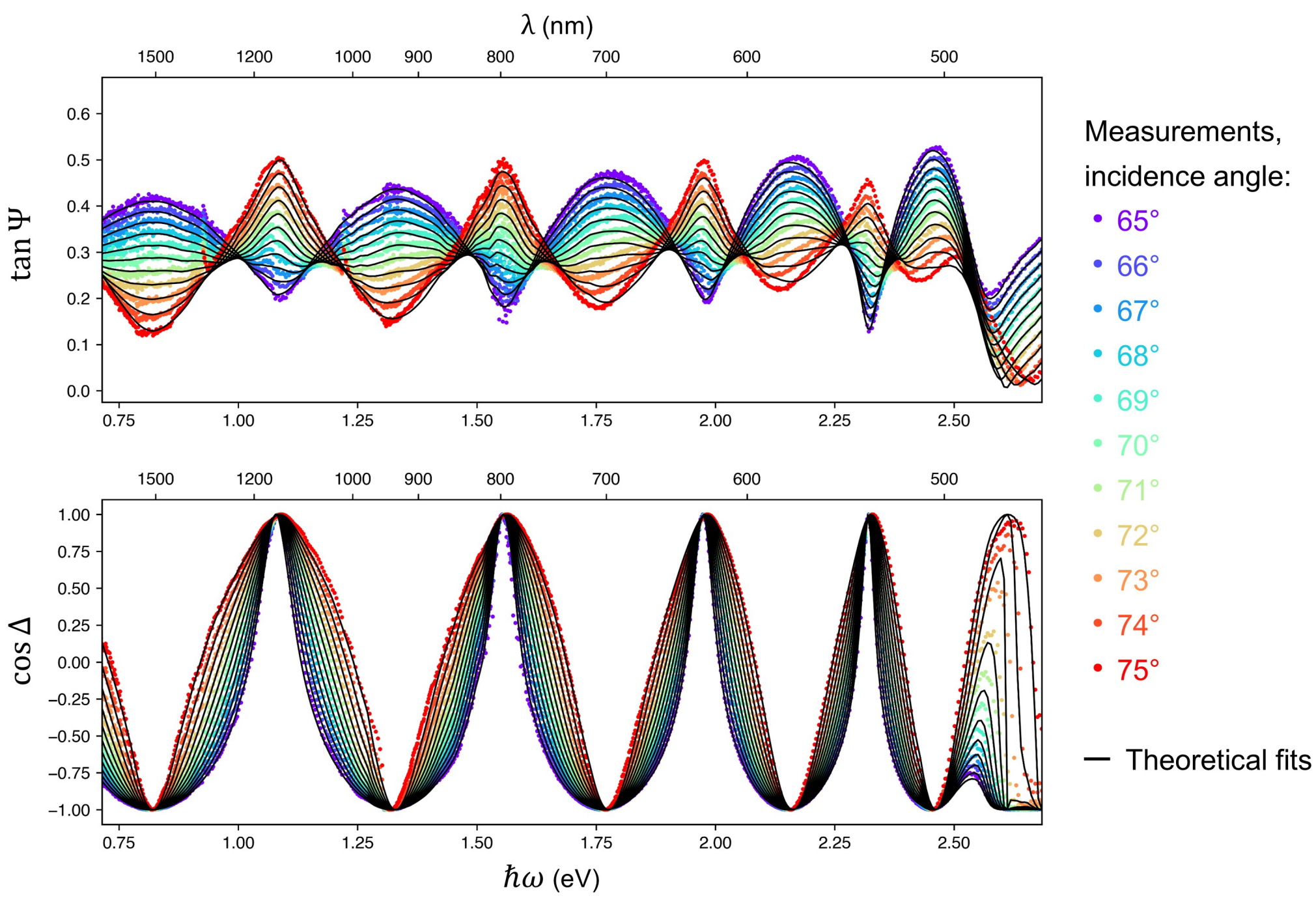

Figure S4 Ellipsometry data ($\tan\Psi$ and $\cos\Delta$ in the top and bottom plots, respectively) measured from the AlInP layer bonded to glass. The colors correspond to different incidence angles, and the black solid lines show the theoretical fits. The fitted AlInP thickness is 406.5 nm. The extracted optical constants are shown in Fig. S5 and Table S1.

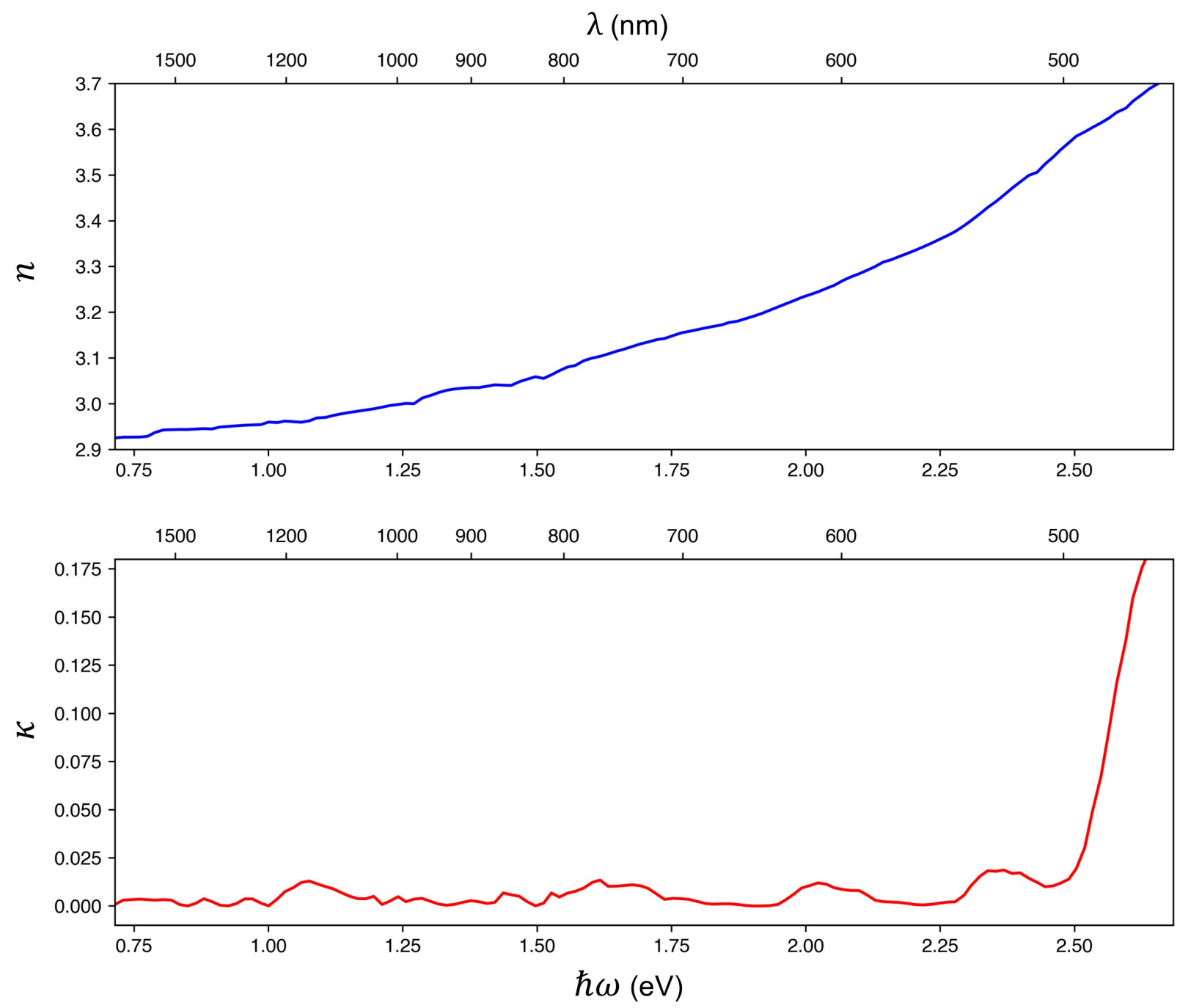

Figure S5 Real and imaginary parts of the refractive index (n and κ in the top and bottom plot, respectively) obtained by fitting the ellipsometry data in Fig. S4.

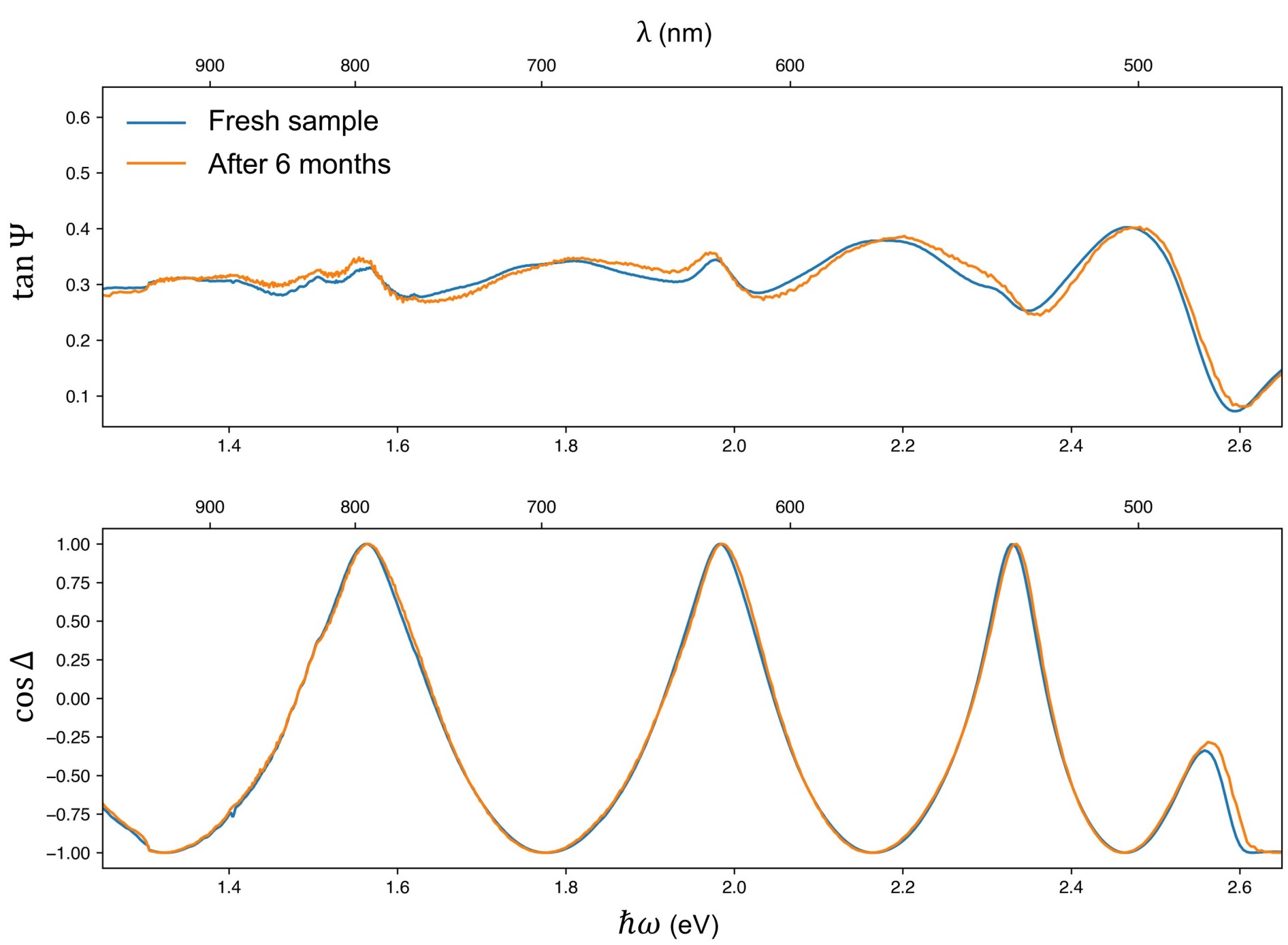

Figure S6 Ellipsometry data for the AlInP layer at the incidence angle of 70°, measured directly after exposing the AlInP surface by wet etching of GaAs (blue curve) and six months later (orange curve).

Table S1 Optical constants of AlInP extracted from the ellipsometry measurements

λ (μm)	n	κ	λ (μm)	n	κ	λ (μm)	n	κ
0.461923	3.737529	0.203645	0.612895	3.244523	0.011967	0.910526	3.033868	0.001851
0.464276	3.709873	0.193388	0.617601	3.238277	0.010539	0.921114	3.032099	0.000780
0.467021	3.699110	0.183131	0.622307	3.232239	0.009280	0.930917	3.029303	0.000353
0.469766	3.688347	0.184009	0.627012	3.224933	0.005994	0.941191	3.024581	0.000931
0.472119	3.676113	0.175904	0.631718	3.218164	0.003128	0.948913	3.019859	0.001921
0.475256	3.661155	0.159931	0.636424	3.211395	0.000733	0.964015	3.012183	0.003905
0.477609	3.646198	0.138007	0.641521	3.204119	0.000148	0.975550	2.999990	0.003515
0.480746	3.637054	0.116083	0.646227	3.197399	0.000000	0.987085	3.000693	0.002188
0.483491	3.624424	0.091186	0.651717	3.191061	0.000000	0.998620	2.998258	0.004801
0.486236	3.614155	0.067694	0.656815	3.185739	0.000260	1.011803	2.995823	0.002377
0.489373	3.603886	0.049022	0.661912	3.180324	0.000733	1.023338	2.992306	0.000753
0.492118	3.594324	0.030350	0.667402	3.177624	0.001108	1.036521	2.988790	0.004973
0.495255	3.584763	0.019306	0.672500	3.172271	0.001070	1.049703	2.986183	0.003726
0.498000	3.570456	0.013912	0.678382	3.168865	0.000910	1.062886	2.983576	0.003729
0.501137	3.554799	0.011789	0.683872	3.165516	0.001290	1.077717	2.980782	0.005134
0.503882	3.539284	0.010397	0.689754	3.161676	0.002364	1.090900	2.977988	0.006797
0.507019	3.523768	0.009968	0.695636	3.157927	0.003420	1.107379	2.973969	0.008979
0.510156	3.505622	0.012287	0.701126	3.154797	0.003733	1.120561	2.969950	0.010030
0.513293	3.499384	0.014329	0.707400	3.148660	0.003914	1.137040	2.968929	0.011499
0.516822	3.484690	0.017182	0.713674	3.142524	0.003409	1.151871	2.962587	0.012887
0.519959	3.471538	0.016837	0.719556	3.140073	0.006071	1.168349	2.959484	0.012144
0.523489	3.455633	0.018612	0.726223	3.135002	0.009060	1.184828	2.960723	0.009487
0.526626	3.442155	0.017983	0.732497	3.130750	0.010436	1.202955	2.961961	0.007356
0.530155	3.429062	0.018233	0.739163	3.125057	0.010964	1.219433	2.958728	0.003627
0.533292	3.415788	0.015533	0.745829	3.119363	0.010614	1.239208	2.959822	0.000000
0.537213	3.400399	0.010595	0.752496	3.114553	0.010232	1.257334	2.954300	0.001459
0.540350	3.388946	0.005501	0.759554	3.108747	0.010196	1.277108	2.953597	0.003601
0.544272	3.376260	0.002125	0.766613	3.103412	0.013403	1.296883	2.952894	0.003633
0.547409	3.368244	0.001977	0.773671	3.099659	0.012172	1.318305	2.951583	0.001191
0.551330	3.359227	0.001427	0.781122	3.094144	0.009323	1.339727	2.950289	0.000030
0.554859	3.351144	0.000844	0.788964	3.083664	0.007608	1.362797	2.948994	0.000382
0.558781	3.342963	0.000480	0.796415	3.080124	0.006638	1.385867	2.944709	0.002278
0.562702	3.335181	0.000714	0.804258	3.072152	0.004600	1.408937	2.945474	0.003715
0.566623	3.328028	0.001332	0.812100	3.063102	0.006707	1.433655	2.944534	0.001310
0.570545	3.321295	0.001818	0.819943	3.055240	0.001369	1.458373	2.943631	0.000000
0.574466	3.314384	0.002006	0.828178	3.058870	0.000051	1.484739	2.943711	0.000684
0.578388	3.309321	0.002219	0.836805	3.053482	0.002204	1.512753	2.943179	0.003009
0.582309	3.299698	0.002978	0.845040	3.048094	0.004994	1.540766	2.942648	0.003262
0.586622	3.291310	0.005802	0.854059	3.039879	0.005781	1.570428	2.937282	0.003014
0.590544	3.284106	0.007958	0.862686	3.040474	0.006755	1.600089	2.928512	0.003262
0.595249	3.276593	0.008018	0.872097	3.041266	0.001829	1.631399	2.927015	0.003509
0.599171	3.269245	0.008541	0.881116	3.038220	0.001283	1.664356	2.926889	0.003254
0.603876	3.258807	0.009411	0.890919	3.035173	0.002164	1.698961	2.926763	0.002999
0.608190	3.252129	0.011258	0.900331	3.035211	0.002724	1.735214	2.925104	0.000732

V Experimental setup

The schematic diagram of our experimental setup is presented in Fig. S7. In the SHG measurements, we used a femtosecond laser (Spectra-Physics, InSight DS+) with wavelength tunable in the range of $\lambda = 680$–$1300\,\mathrm{nm}$, pulse duration τ_p of about $100\,\mathrm{fs}$, and repetition rate $f_{rep} = 80\,\mathrm{MHz}$. The power level of the pump beam was controlled with a half-wave plate (HWP) and a linear polarizer (LP; in this case: a polarizing cube beamsplitter). Another HWP was used to control the polarization, and a long-pass filter (LPF) blocked any unwanted short-wavelength radiation propagating with the pump.

In the transmission measurements, we used a quartz tungsten-halogen lamp (Thorlabs, QTH10/M), emitting in a broad spectral range of $\lambda = 200$–$2000\,\mathrm{nm}$. The beam was collimated, spatially filtered, and polarized using another LP. A flip-mirror was used to switch between the two light sources.

The rest of the setup was similar for both the nonlinear and transmission measurements. The light was directed to a galvo mirror (GM), whose tilt was controlled with an applied voltage. The reflected beam was focused by an aspheric lens ($f = 50\,\mathrm{mm}$) in the back-focal plane of an objective (OBJ1, OptoSigma, PAL-20-NIR-HR-LC00, NA $= 0.45$). The sample was mounted on a manual translation stage combined with a goniometer and positioned in the focus of OBJ1. Such a configuration transfers the GM tilt into the incidence angle θ_y on the sample. To scan the incidence angle, we used a periodic ramp-type electric signal from a function generator to drive GM. The transmitted light (or the SHG emission from the sample) was collected by another objective (OBJ2, Zeiss, N-Achroplan 63$\times$, NA $= 0.95$) and imaged on the entrance slit of the imaging spectrometer (HORIBA, iHR320) with a sCMOS camera (Hamamatsu, ORCA-Quest). The last filter before the slit was either a short-pass filter (SPF) for measuring the SHG signal in the visible wavelength range, or another long-pass filter to measure the transmission in the infrared range. For transmission measurements in the visible range, all filters were removed. In the imaging part of the setup, we switched between two different configurations of lenses and mirrors (see the scheme in Fig. S7) to perform either real-space or k-space imaging. At the same time, by rotating the grating inside the spectrometer (selecting either the 0th or the 1st diffraction order) and modifying the width of the entrance slit, we switched between the wide-field and spectrally resolved imaging.

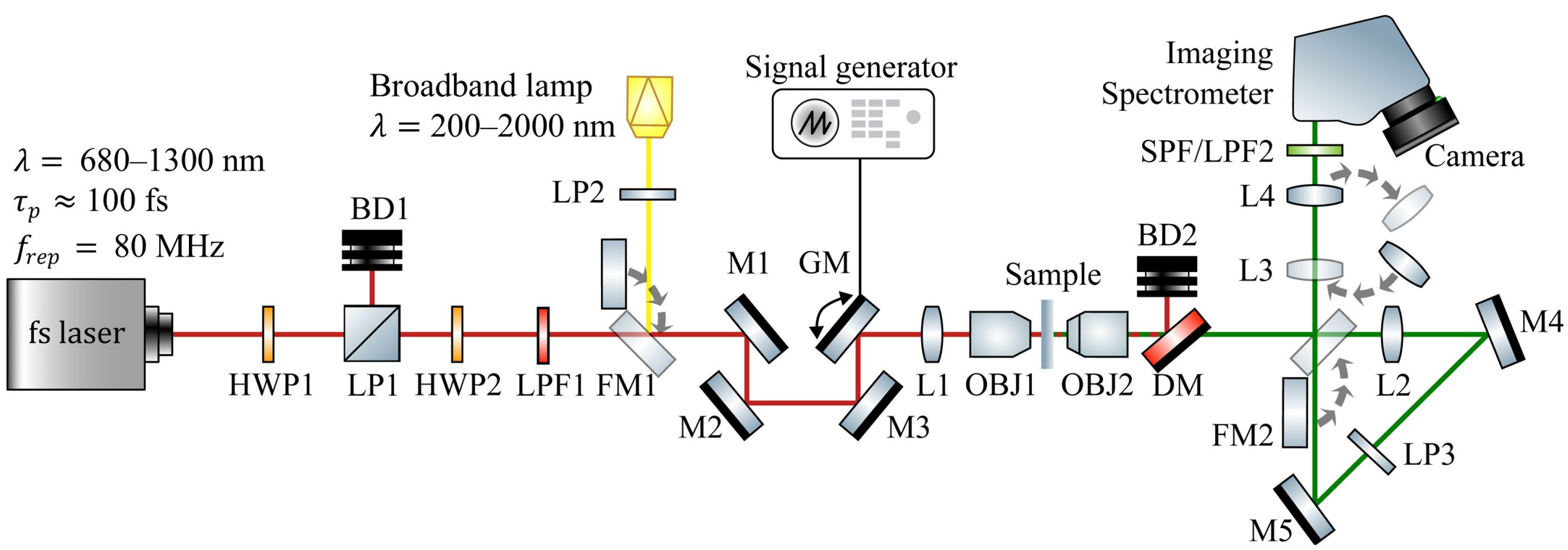

Figure S7 Schematic of the used experimental setup. HWP - half-wave plate, LP - linear polarizer, BD - beam dump, LPF - long-pass filter, M - mirror, FM - flip mirror, GM - galvo mirror, L - lens, OBJ - objective, DM - dichroic mirror, SPF - short-pass filter.

VI Power dependence of the SHG signal

For both samples, we measured the SHG signal as a function of the average pump power. The measured data are plotted on a log-log scale in Fig. S8. We fitted these plots with a linear function $\log(\mathscr{P}_{\text{SHG}}) \propto \alpha \log(\mathscr{P}_{\text{pump}})$. In both cases, the fitted slope is $\alpha \approx 2$, indicating a quadratic power dependence, which is a key characteristic of SHG. Furthermore, the dependence did not show saturation up to $\mathscr{P}_{\text{pump}} = 250\,\text{mW}$, which was the highest power used in the experiments.

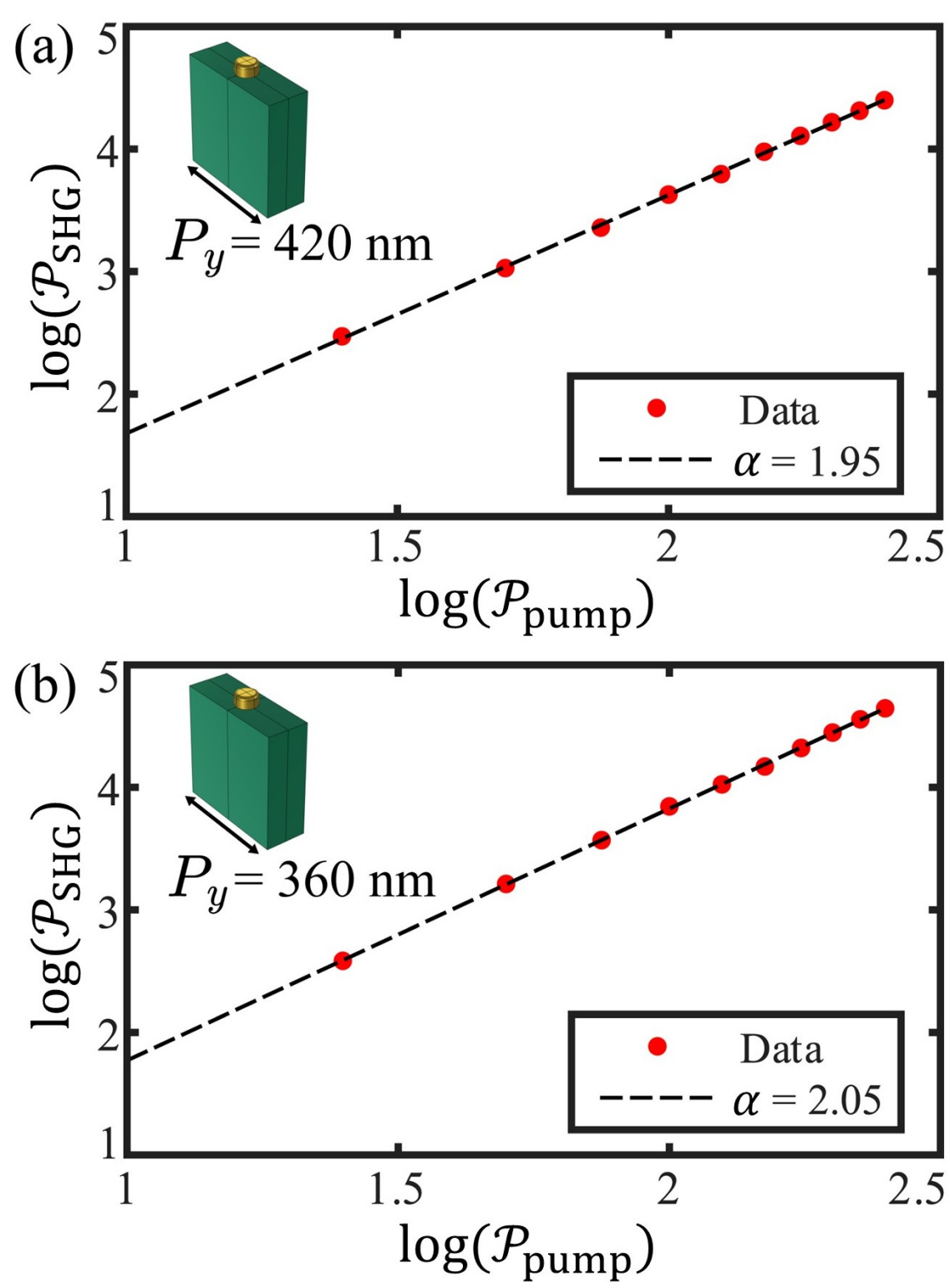

Figure S8 Dependence of the measured SHG signal $\mathscr{P}_{\text{SHG}}$ on the average pump power $\mathscr{P}_{\text{pump}}$, plotted on a double-logarithmic scale, for samples A ($P_y = 420\,\text{nm}$) and B ($P_y = 360\,\text{nm}$) in panels (a) and (b), respectively. The red dots show the measured data points, while the dashed black lines show the linear fit to the data, with α being the slope of the fit. The units of $\mathscr{P}_{\text{SHG}}$ and $\mathscr{P}_{\text{pump}}$ are counts/s and mW, respectively.

VII Data and labels from Fig. 3a-b shown separately

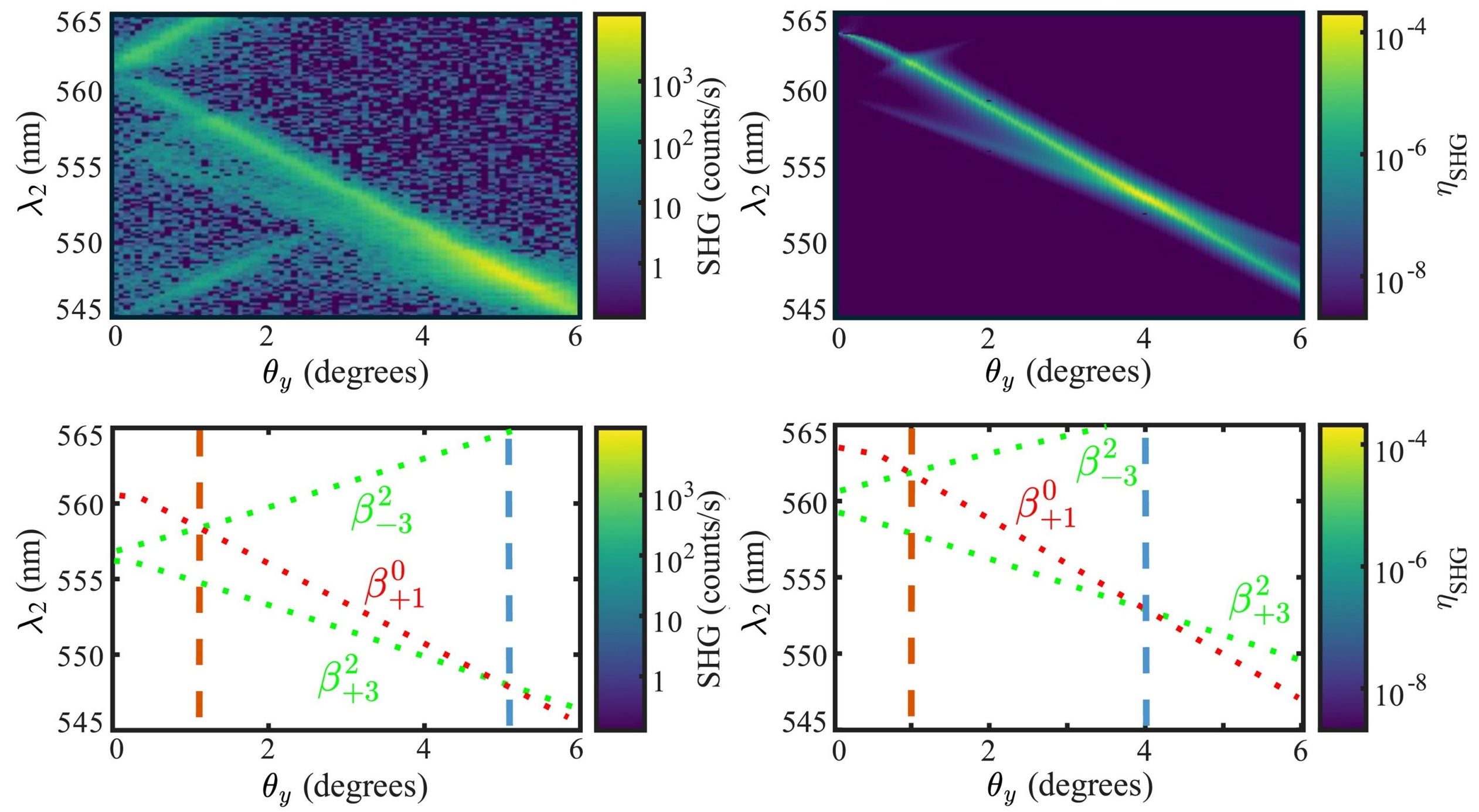

Figure S9 The same data and labels as in Fig. 3a and b, but shown in separate panels.

VIII Extracting the Q factor from the SHG map

Figure S10 demonstrates the procedure that we used to extract the Q factors from the measured SHG powers as functions of λ_2 and θ_y. First, we compensated for the linear angular dispersion of the GMRs – we fitted the data with functions $\lambda_2 = A\theta_y + B$ (see the red and yellow lines in Fig. S10a) using the magnitude of the SHG signal as weights. Next, we converted the wavelength axis to $\lambda_2' = \lambda_2 - (A\theta_y + B)$ and linearly interpolated the data over a dense regular grid in the λ_2'-θ_y parameter space (see Fig. S10b,c). Finally, we integrated the obtained 2D maps along the θ_y-axis to get the averaged GMR lineshapes, which we fitted with modified Lorentzian functions (see Fig. S10d,e). In the case of the first peak, corresponding to the pump-resonant β^0_{+1} mode, we fitted the data using the sum of a squared Lorentzian curve and a linear function, $C/(1+(\lambda_2')^2/\gamma^2)^2 + A\lambda_2' + B$, while for the second peak, i.e., the second-harmonic mode β^2_{+3}, we used a similar function but without squaring the Lorentzian curve, $C/(1+(\lambda_2')^2/\gamma^2) + A\lambda_2' + B$. The fitted parameter 2γ yielded the full width at half maximum ($\Delta\lambda = 2\gamma$), which we used to calculate the Q factor from $Q = \lambda_1/\Delta\lambda = 2\lambda_2/\Delta\lambda$ for the β^0_{+1} mode and $Q = \lambda_2/\Delta\lambda$ for the β^2_{+3} mode.

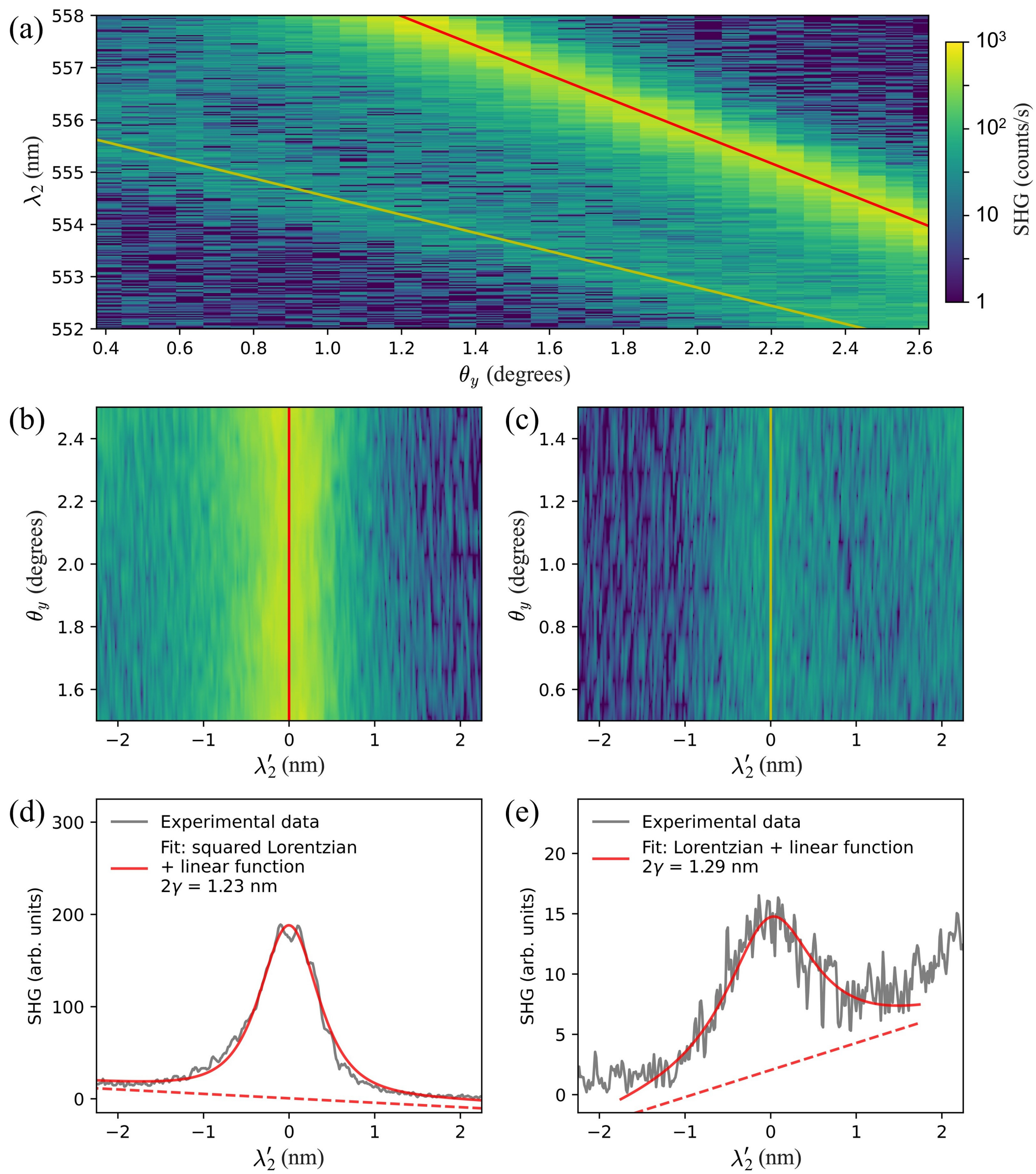

Figure S10 Illustration of the procedure to extract the GMR's Q factors from the measured dependence of the SHG on λ_2 and θ_y. (a) Part of the original data (the same as in Figs. 2c and 3a), with the GMR dispersion lines fitted with linear functions (red and yellow lines for the β^0_{+1} and β^2_{+3} modes, respectively). (b, c) The same data as in (a), but compensated for the spectral shifts of the GMR peaks and linearly interpolated over a dense regular grid. The values displayed in the plots correspond to the color bar shown in (a); note the logarithmic scale. (d, e) Averaged GMR peaks (β^0_{+1} and β^2_{+3} modes, respectively) obtained by integrating the data in (b, c) along the θ_y-axis. The Q factors are extracted by fitting the data with functions $C/(1+(\lambda_2')^2/\gamma^2)^2+A\lambda_2'+B$ and $C/(1+(\lambda_2')^2/\gamma^2)+A\lambda_2'+B$, which yields the spectral widths of the peaks $\Delta\lambda=2\gamma$. We obtained $Q=2\lambda_2/\Delta\lambda\approx 900$ for the β^0_{+1} mode and $Q=\lambda_2/\Delta\lambda\approx 430$ for the β^2_{+3} mode.

IX Numerical simulations

In our numerical simulations, we used the commercial finite-element method software COMSOL Multiphysics. The metasurfaces were modeled as a single unit cell with periodic boundary conditions in the lateral directions (x and y), in which we used Floquet wavevector to model the optical response at oblique angles. Along the z-direction, the geometry was terminated with a scattering boundary condition emulating the far-field radiation condition. The metasurface geometry contained the 410 nm thick AlInP layer on a glass substrate (SCHOTT BOROFLOAT 33), with disc-shaped gold nanoparticles on the top surface (diameter 80 nm, height 40 nm). We used the optical constants of gold and glass from Refs.[2,3] and the glass manufacturer's data sheet, respectively, whereas the optical constants of AlInP were based on our ellipsometric data.

The simulations were performed in the frequency domain. To obtain the band diagrams in Fig. 1c-d, and the electric field distributions in Figs. 1e-f and 3f, we used the eigenfrequency analysis. To simulate SHG (Fig. 3b, d, and e), we performed scattered-field simulations in two steps, first at the fundamental wavelength and second at the SHG wavelength[4–6]. In the first step, the metasurface was illuminated by a plane wave incident from the substrate side at an angle corresponding to the free-space incidence angle θ_y. The calculated electric field distribution was used to evaluate the nonlinear polarization density. In the second step, the nonlinear polarization density was used as a source of the SH field, and the resulting SHG emission from the metasurface into the far-field was calculated through full-wave numerical simulation performed at the SH frequency. We assumed that the second-order susceptibility is nonzero only in the AlInP layer, with the magnitude of $d_{36} = 39\,\mathrm{pm/V}$[7] and the $\chi^{(2)}$-tensor elements corresponding to the zinc-blende crystal lattice after appropriate transformation (see Section X). The SHG intensity $I(\lambda_2)$ was evaluated at a distance of 1 μm from the metasurface. The conversion efficiency was obtained from

$$\eta_{\mathrm{SHG}} = \frac{I(\lambda_2)}{I(\lambda_1)}, \tag{1}$$

with the incident fundamental wave intensity $I(\lambda_1)$ of $38.8\,\mathrm{MW/cm^2}$ corresponding to the experimental conditions.

X AlInP crystal orientation and the $\chi^{(2)}$ tensor

Similar to many III-V semiconductors, AlInP has a zinc-blende-type crystal structure, belonging to the $\bar{4}3m$ point group. In the conventional lattice orientation (with the [100], [010], and [001] crystallographic directions aligned with the x, y, and z directions), and assuming that Kleinman symmetry holds, the $\chi^{(2)}$ tensor has only one independent nonzero element with $\chi^{(2)}_{xyz} = \chi^{(2)}_{yxz} = \chi^{(2)}_{zxy} = \chi^{(2)}_{zyx} = \chi^{(2)}_{xzy} = \chi^{(2)}_{yzx} = 2d_{36}$[8]. The nonlinear polarization is then given by

$$\mathbf{P}^{(2)} = 2\varepsilon_0 d_{36} \begin{bmatrix} 2E_yE_z \\ 2E_xE_z \\ 2E_xE_y \end{bmatrix}, \tag{2}$$

where E_i are the Cartesian components of the electric field vector.

In our samples, the AlInP crystal lattice is rotated by 45° in the xy-plane with respect to the xyz frame of the metasurface and experimental setup. The rotation matrix is

$$\mathbf{R} = \begin{bmatrix} \cos 45^\circ & -\sin 45^\circ & 0 \\ \sin 45^\circ & \cos 45^\circ & 0 \\ 0 & 0 & 1 \end{bmatrix}. \tag{3}$$

The $\chi^{(2)}$ tensor should be transformed according to[9]

$$\chi^{(2)}_{ijk} = \sum_{I=1}^{3} \sum_{J=1}^{3} \sum_{K=1}^{3} \chi^{(2)}_{IJK} R_{iI} R_{jJ} R_{kK}, \tag{4}$$

which gives $\chi^{(2)}_{yyz} = \chi^{(2)}_{yzy} = \chi^{(2)}_{zyy} = -\chi^{(2)}_{xxz} = -\chi^{(2)}_{xzx} = -\chi^{(2)}_{zxx}$. Hence, the nonlinear polarization in AlInP is

$$\mathbf{P}^{(2)} = 2\varepsilon_0 d_{36} \begin{bmatrix} -2E_xE_z \\ 2E_yE_z \\ -E_x^2 + E_y^2 \end{bmatrix}. \tag{5}$$

In our experiments, the pump is always y-polarized and the incidence angle θ_y lies in the yz-plane. Ignoring the effect of near-field depolarization due to the presence of plasmonic nanoparticles, the measured SHG signal can be assumed to originate mainly from the $\chi^{(2)}_{yyz}$ and $\chi^{(2)}_{zyy}$ elements. Since both the pump and SHG fields contain mainly the y- and z-components, they are efficiently coupled to the TM-polarized GMRs.

XI Simulated SHG response for sample B

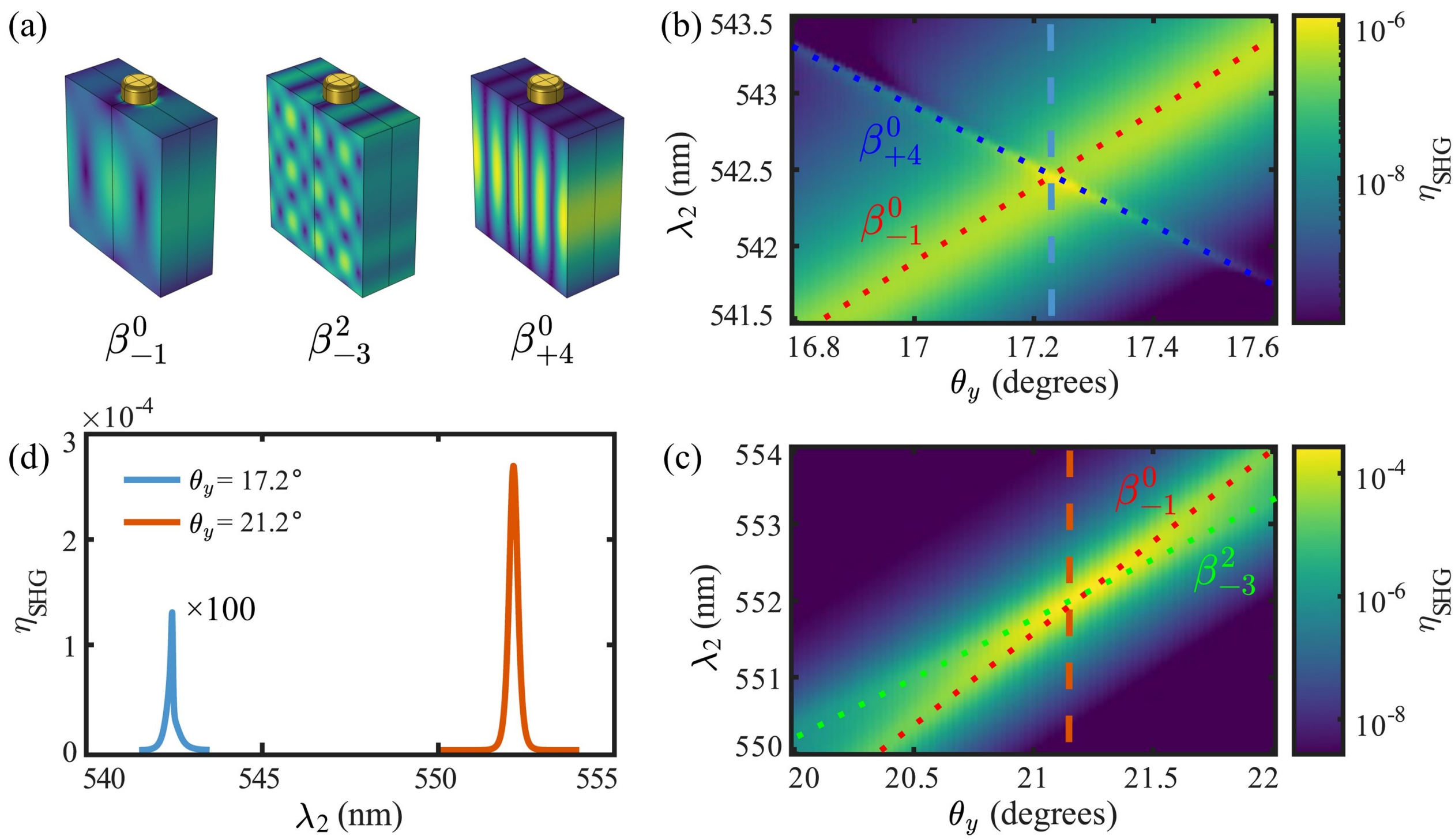

Figure S11 Simulated SHG response for sample B ($P_y = 360\,\mathrm{nm}$). (a) Electric field distributions ($|\mathbf{E}|$) of the GMRs that contribute to the SHG enhancement, calculated at $\theta_y = 0$ using the eigenfrequency analysis in COMSOL. (b, c) Simulated SHG conversion efficiency (η_{SHG}) at the doubly resonant conditions as a function of the incidence angle (θ_y) and SHG wavelength (λ_2). (d) Vertical crosscuts of the plots in (b) and (c) (blue and orange curves, respectively) comparing the peaks at the doubly resonant SHG enhancement. The peak at $\theta_y = 17.2°$ (blue) is multiplied by a factor of 100 to make it visible. The maximum SHG enhancement at $\theta_y = 21.2°$ (orange) is 200 times greater than the maximum enhancement at $\theta_y = 17.2°$.

Figure S11 shows the results of the SHG simulations for sample B ($P_y = 360\,\mathrm{nm}$). The simulated responses agree well with the experimental results (see Fig. 4 in the main article). In particular, the doubly resonant conditions occur at nearly the same parameter values as in the experiment. Furthermore, the SHG enhancement follows exactly the same trend, becoming significantly stronger for the co-propagating fundamental and SH GMRs.

XII TE-polarized guided-mode resonances

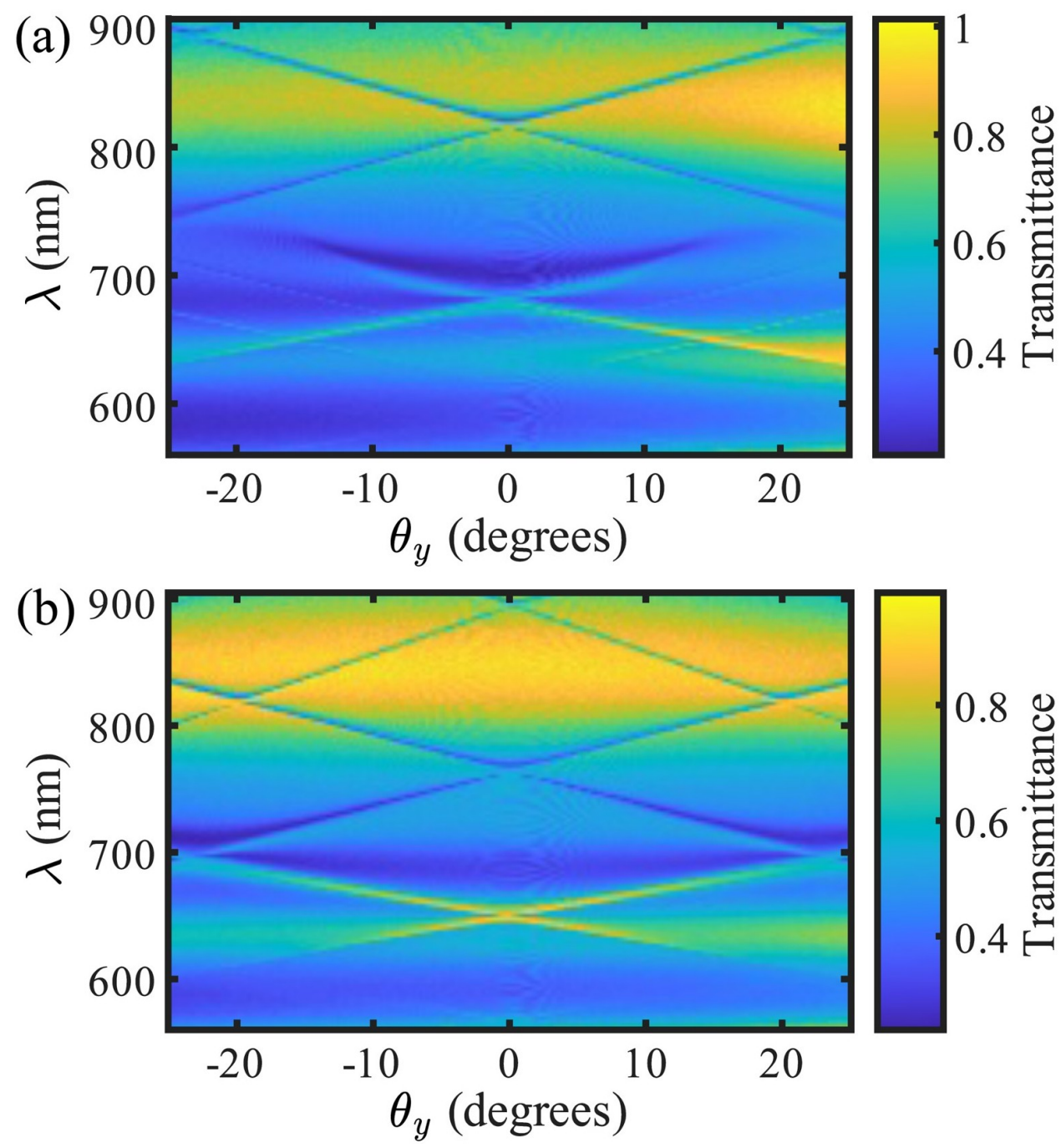

Figure S12 Transmission of broadband x-polarized light measured as a function of the wavelength and incidence angle for (a) sample A ($P_y = 420\,\text{nm}$) and (b) sample B ($P_y = 360\,\text{nm}$), revealing the dispersive high-Q TE-polarized GMRs.

In this work, we focused on the TM-polarized GMRs excited by the y-polarized incident light, because only these modes have the nonzero electric field components (E_y and E_z) that can couple to the nonzero elements of the $\chi^{(2)}$ tensor. However, the studied samples also exhibit transverse electric (TE) GMRs. Although they do not contribute to the SHG enhancement, they give rise to narrow features in the transmission spectra measured under illumination by the x-polarized light, as shown in Fig. S12. Based on their spectral widths, the Q factors can be estimated at about 10^3, similar to the TM GMRs.

References

[1] A. Holmes, M. Islam, R. Chelakara, F. Ciuba, R. Dupuis, M. Ries, E. Chen, S. Maranowski and N. Holonyak Jr, *Applied Physics Letters*, 1995, **66**, 2831–2833.

[2] M. N. Polyanskiy, *Scientific Data*, 2024, **11**, 94.

[3] P. B. Johnson and R.-W. Christy, *Physical Review B*, 1972, **6**, 4370.

[4] G. Bachelier, J. Butet, I. Russier-Antoine, C. Jonin, E. Benichou and P.-F. Brevet, *Physical Review B*, 2010, **82**, 235403.

[5] R. Kolkowski, L. Petti, M. Rippa, C. Lafargue and J. Zyss, *ACS Photonics*, 2015, **2**, 899–906.

[6] R. Kolkowski, J. Szeszko, B. Dwir, E. Kapon and J. Zyss, *Laser & Photonics Reviews*, 2016, **10**, 287–298.

[7] I. Shoji, T. Kondo and R. Ito, *Optical and Quantum Electronics*, 2002, **34**, 797–833.

[8] R. W. Boyd, *Nonlinear optics*, Academic Press, San Diego, 2020.

[9] E. Jatirian-Foltides, J. Escobedo-Alatorre, P. Márquez-Aguilar, H. Hardhienata, K. Hingerl and A. Alejo-Molina, *Revista Mexicana de Física E*, 2016, **62**, 5–13.
